# Crystal structures of the Lewis acid–base adducts {[(C_6_H_11_)_2_N]_3_Ti–*LB*}^+^[MeB(C_6_F_5_)_3_]^−^·1.5C_7_H_8_; LB = (C_6_H_5_)_3_PO (1), *p*-F—C_6_H_4_CN (2)

**DOI:** 10.1107/S2056989022007952

**Published:** 2022-08-16

**Authors:** Kerstin Fitschen, Nils Frerichs, Zainab Yusufzadeh, Marc Schmidtmann, Rüdiger Beckhaus

**Affiliations:** a Carl von Ossietzky Universität Oldenburg, Fak. V, Institut für Chemie, Carl-von-Ossietzky-Strasse 9-11, D-26129 Oldenburg, Germany; Vienna University of Technology, Austria

**Keywords:** crystal structure, titanium, lewis acidity, disorder, C—H⋯F hydrogen-bonding

## Abstract

The reaction of [(C_6_H_11_)_2_N]_3_Ti—CH_3_ with the Lewis acid B(C_6_F_5_)_3_, followed by addition of the Lewis bases (C_6_H_5_)_3_PO and *p*-F—C_6_H_4_CN led to the complex salts tris­(di­cyclo­hexyl­amido)(tri­phenyl­phosphine oxide)titanium methyl­tris(penta­fluoro­phen­yl)borate toluene sesquisolvate, [Ti(C_12_H_22_N)_3_(C_18_H_15_OP)](C_19_H_3_F_15_B)·1.5C_7_H_8_, (**1**), and tris­(di­cyclo­hexyl­amido)(4-fluoro­benzo­nitrile)­titanium methyl­tris­(penta­fluoro­phen­yl)borate toluene sesquisolvate, [Ti(C_12_H_22_N)_3_(C_7_H_4_FN)](C_19_H_3_F_15_B)·1.5C_7_H_8_, (**2**), both crystallizing with 1.5 equivalents of toluene solvent mol­ecules. The Lewis acid–base adducts (**1**) and (**2**) can be described by dative donor bonds. The packing of the complex cations, anions and solvent mol­ecules in the crystal structure is consolidated by an intricate three-dimensional network of non-classical C—H⋯F inter­actions. Disorder of some of the cyclo­hexyl groups and the toluene solvent mol­ecules is observed.

## Abstract 2

In the mol­ecular structures of the two title compounds, the central titanium(IV) atoms are coordinated in a distorted tetra­hedral fashion. Thereby, the titanium atom is stabilized by the Lewis bases tri­phenyl­phosphine oxide (**1**) and *p*-fluoro­benzo­nitrile (pFBN) (**2**) with dative O—Ti and N—Ti bonds, respectively.

## 1. Chemical context

Highly electrophilic *d* ^0^ cations of group 4 metals are known as strong Lewis acids (Bochmann, 2010 ▸). Among various other applications, Lewis acids are used in reactions to activate small mol­ecules or in the catalysis of chemical reactions (Corma & García, 2003 ▸). The use of a Lewis acid in a catalytic reaction requires knowledge of its strength (Greb, 2018 ▸). There are now many different ways of determining the Lewis acidity of a compound. The best known is the Gutmann–Beckett method (Mayer *et al.*, 1975 ▸). Another possibility is the use of *para*-fluoro­benzo­nitrile (Künzler *et al.*, 2019 ▸). In this context, we report here syntheses and crystal structures of the Lewis acid-base adducts derived from Ti^IV^ as the Lewis acid, {[(C_6_H_11_)_2_N]_3_TiOP(C_6_H_5_)_3_}^+^[H_3_CB(C_6_F_5_)_3_]^−^·1.5C_7_H_8_ (**1**) and {[(C_6_H_11_)_2_N]_3_TiNC_7_H_4_F}^+^[H_3_CB(C_6_F_5_)_3_]^−^·1.5C_7_H_8_ (**2**). 
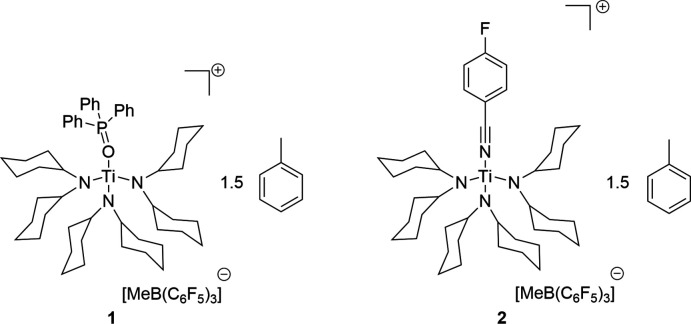


## 2. Structural commentary

**Figure 1:**
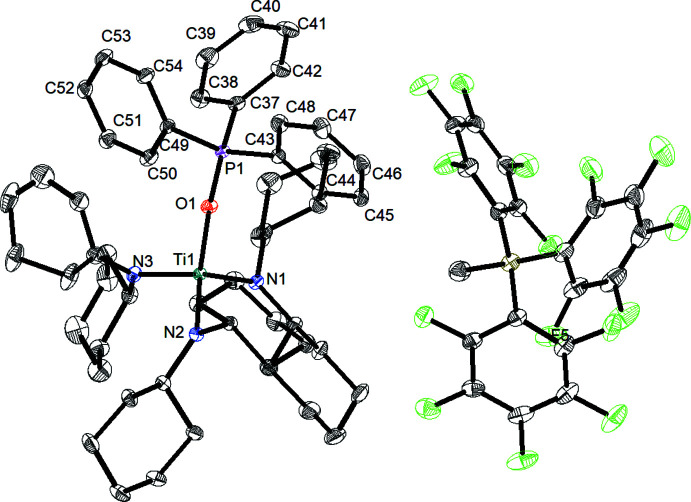
Mol­ecular structure of compound (**1**). Displacement ellipsoids correspond to the 50% probability level. For clarity, only the major components of disordered parts are shown, and H atoms as well as solvent mol­ecules have been omitted.

In compound (**1**), the titanium(IV) atom is coordinated by three N atoms and one O atom in a slightly distorted tetra­hedral fashion (Fig. 1 ▸) with bond angles around titanium(IV) ranging from 105.94 (4) to 112.06 (4)°. The Ti—O bond length [1.9782 (8) Å] is in the range of a single bond (1.99 Å), similar to titanium–tri­phenyl­phosphane adducts (Pyykkö & Atsumi, 2009 ▸; Brock *et al.*, 1985 ▸). The Ti—O—P angle amounts to 170.99 (5)° and thus indicates a bonding situation deviating only slightly from linearity. As a result of the coordination of the tri­phenyl­phosphine oxide ligand to the central titanium atom, the O—P bond [1.5269 (8) Å] is widened compared to the O—P bond in the free phosphine oxide [1.491 Å; Brock *et al.*, 1985 ▸). The angular sums around the nitro­gen atoms reveal a trigonal–planar environment in each case, with values of 359.9° for N1, 359.7° for N2 and 360.0° for N3 (using only the major component of the disordered ligand for calculation).

**Figure 2:**
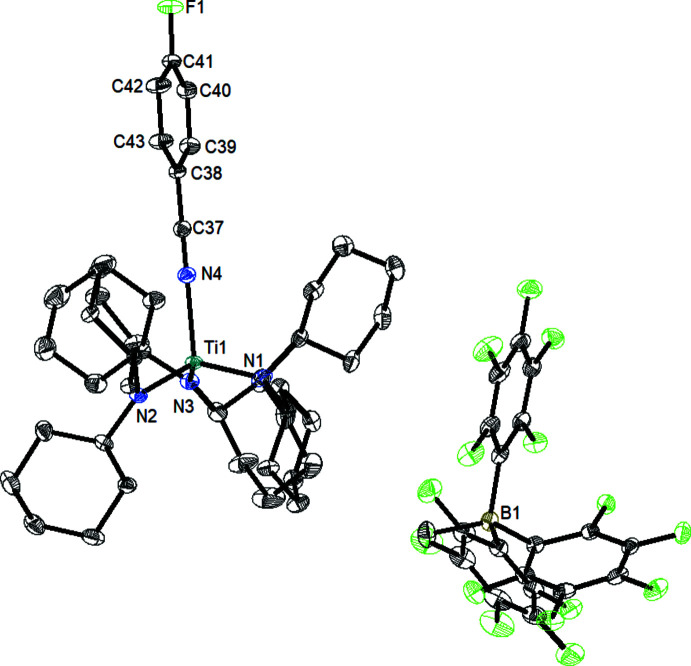
Mol­ecular structure of compound (**2**). Displacement ellipsoids correspond to the 50% probability level. For clarity, only the major components of disordered parts are shown, and H atoms as well as solvent mol­ecules have been omitted.

In compound (**2**), the titanium(IV) atom is coordinated by four N atoms in a likewise slightly distorted tetra­hedral fashion (Fig. 2 ▸) with bond angles around titanium(IV) ranging from 104.66 (4) to 113.62 (4)°. The Ti1—N4 bond length of 2.1608 (9) Å to the N≡C group of the *para*-fluoro­benzo­nitrile ligand is in the range of a dative bond (Pyykkö & Atsumi, 2009 ▸), and by far greater than the other three Ti—N bonds (≃ 1.89 Å), which are in the range of shortened single bonds. The Ti1—N4—C37 angle [175.04 (9)°] and the N4—C37—C38 angle [179.25 (12)°] indicate a nearly linear arrangement of the Ti—N≡C—C fragment. The trigonal-planar environment of the three nitro­gen atoms of the amido ligands is described by the angle sums N1 = 359.3° (using only the major component of the disordered ligand for calculation), N2 = 359.9° and N3 = 360.0°.

## 3. Supra­molecular features

**Figure 3:**
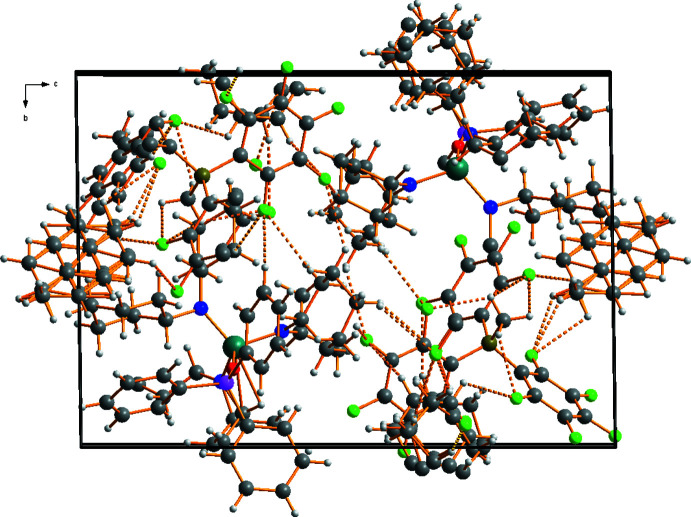
A view along the *a* axis showing the packing of individual mol­ecules in the crystal of (**1**), with C—H⋯·F bonds shown as dashed lines. Both components of the disorder are shown. Colour code: C dark grey, H light grey, N blue, Ti teal, B dark yellow, F bright green, P purple, O red.

Compounds (**1**) and (**2**) each crystallize with 1.5 equivalents of toluene per formula unit, all of them disordered. An intricate three-dimensional network of non-classical C—H⋯F inter­actions is formed in the crystals of (**1**) and (**2**), involving the C—H groups of both cations and solvent mol­ecules as donors, and some F atoms of the [H_3_CB(C_6_F_5_)_3_]^−^ anions as acceptors (Figs. 3 ▸, 4 ▸). Numerical data of these inter­actions are compiled in Tables 1 ▸ and 2 ▸.

## 4. Synthesis and crystallization

All reactions were carried out under a dry nitro­gen atmosphere using Schlenk techniques or in a glove box. Solvents were dried according to standard procedures over Na/K alloy with benzo­phenone as an indicator and distilled under a nitro­gen atmosphere. The cationic titanium complex was synthesized by reacting tris-(di­cyclo­hexyl­amido)­methyl­titanium and tris-penta­fluorphenyl­borane (Adler *et al.*, 2016 ▸). The cationic titanium complex was dissolved in 3 ml of toluene, the respective base added and stirred at room temperature for 16 h. Afterwards, the solvent was removed by evaporation. Single crystals of compound (**1**) and (**2**) were obtained from a saturated solution of toluene at 243 K.

## 5. Refinement

Crystal data, data collection and structure refinement details are summarized in Table 3 ▸. Hydrogen atoms attached to carbon were placed in idealized positions and refined with a riding model. Compound (**1**) shows disorder of one cyclo­hexyl residue (C25 to C30), located at N3, over two sets of sites, with refined site occupancy factors 0.87:0.13. Compound (**2**) shows disorder of two of the six cyclo­hexyl residues (C7–C12; C32–C36), located at the N1 and N3 atoms, respectively, with refined site occupation factors of 0.76:0.24 and 0.85:0.15. No restraints or constraints were applied during the refinement of this kind of disorder. All non-H atoms were refined anisotropically with the exception of the minor components of the disordered parts, which have been refined isotropically. In addition, the toluene solvent mol­ecule in compounds (**1**) and (**2**) are disordered. In (**1**), one toluene mol­ecule (C81–C87) is equally disordered over an inversion centre, and one (C74–C80) over two sets of sites, with refined site occupation factors of 0.764 (4):0.236 (4). In (**2**), one toluene mol­ecule (C63–C69) is disordered over two sets of sites, the site occupancy was constrained to 0.5 for each component. The other toluene mol­ecule (C70–C76) is disordered over two sets of sites with refined occupation factors of 0.29 (3):0.210 (3), both of which are additionally disordered over an inversion centre, resulting in a disorder over four sites.

## Supplementary Material

Crystal structure: contains datablock(s) 1, 2. DOI: 10.1107/S2056989022007952/wm5649sup1.cif

Structure factors: contains datablock(s) 1. DOI: 10.1107/S2056989022007952/wm56491sup2.hkl

Structure factors: contains datablock(s) 2. DOI: 10.1107/S2056989022007952/wm56492sup3.hkl

CCDC references: 2195527, 2195526

Additional supporting information:  crystallographic information; 3D view; checkCIF report

## Figures and Tables

**Figure 4:**
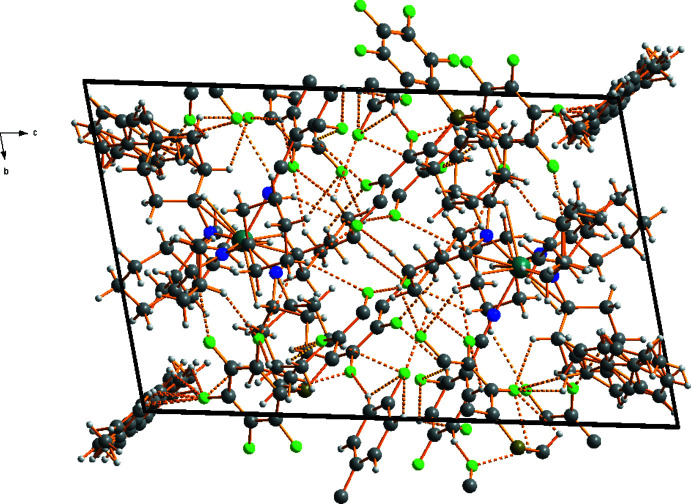
A view along the *a* axis showing the packing of individual mol­ecules in the crystal of (**2**), with C—H⋯·F bonds shown as dashed lines. Both components of the disorder are shown. Colour code: C dark grey, H light grey, N blue, Ti teal, B dark yellow, F bright green.

**Table 1:** Hydrogen-bond geometry (Å, °) for (**1**)

*D*—H⋯*A*	*D*—H	H⋯*A*	*D*⋯*A*	*D*—H⋯*A*
C28*B*—H28*C*⋯F11^i^	0.99	2.27	2.962 (19)	126
C39—H39⋯F4^ii^	0.95	2.50	3.2194 (16)	133
C76*A*—H76*A*⋯F5	0.95	2.45	3.376 (3)	165
C84—H84⋯F1^iii^	0.95	2.45	3.331 (9)	154
C26*B*—H26*D*⋯F6^i^	0.99	2.55	3.446 (13)	150
C35—H35*A*⋯F3^i^	0.99	2.55	3.4738 (18)	155

Symmetry codes: (i) 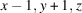 ; (ii) 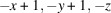 ; (iii) 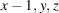 .

**Table 2:** Hydrogen-bond geometry (Å, °) for (**2**)

*D*—H⋯*A*	*D*—H	H⋯*A*	*D*⋯*A*	*D*—H⋯*A*
C62—H62*A*⋯F6	0.98	2.35	2.9753 (17)	121
C62—H62*C*⋯F16	0.98	2.38	2.9931 (16)	120
C9*A*—H9*AB*⋯F2^i^	0.99	2.53	3.1938 (17)	124
C71*B*—H71*B*⋯F14^i^	0.95	2.58	3.176 (5)	121
C17—H17*B*⋯F4^ii^	0.99	2.50	3.2834 (15)	136
C34*A*—H34*A*⋯F16	0.99	2.45	3.296 (2)	143
C43—H43⋯F8^iii^	0.95	2.55	3.2229 (13)	128
C75—H75⋯F14^iv^	0.95	2.47	3.212 (6)	135
C39—H39⋯F11^v^	0.95	2.47	3.3904 (13)	163
C40—H40⋯F6^v^	0.95	2.43	3.3413 (13)	160
C66*B*—H66*B*⋯F11^iii^	0.95	2.49	3.372 (4)	154
C5—H5*B*⋯F10^v^	0.99	2.52	3.4028 (14)	149
C16—H16*B*⋯F9^iii^	0.99	2.56	3.4280 (14)	147

Symmetry codes: (i) 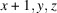 ; (ii) 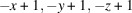 ; (iii) 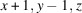 ; (iv) 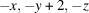 ; (v) 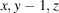 .

**Table 3:** Experimental details

	(**1**)	(**2**)
Crystal data		
Chemical formula	[Ti(C_12_H_22_N)_3_(C_18_H_15_OP)](C_19_H_3_F_15_B)·1.5C_7_H_8_	[Ti(C_12_H_22_N)_3_(C_7_H_4_FN)](C_19_H_3_F_15_B)·1.5C_7_H_8_
*M*_r_	1532.31	1375.15
Crystal system, space group	Triclinic, *P* 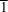	Triclinic, *P* 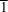
Temperature (K)	100	100
*a*, *b*, *c* (Å)	13.8643 (5), 14.3017 (5), 20.0502 (7)	12.1151 (5), 13.2796 (6), 21.1662 (10)
α, β, γ (°)	86.4852 (13), 80.0328 (13), 73.6493 (12)	77.9160 (19), 88.8857 (18), 87.9827 (18)
*V* (Å^3^)	3756.9 (2)	3327.5 (3)
*Z*	2	2
Radiation type	Mo *K*α	Mo *K*α
μ (mm^−1^)	0.22	0.22
Crystal size (mm)	0.15 × 0.08 × 0.07	0.32 × 0.30 × 0.08

Data collection		
Diffractometer	Bruker Photon III CPAD	Bruker APEXII CCD
Absorption correction	Multi-scan (*SADABS*; Krause *et al.*, 2015 ▸)	Multi-scan (*SADABS*; Krause *et al.*, 2015 ▸)
*T*_min_, *T*_max_	0.958, 1.000	0.963, 1.000
No. of measured, independent and observed [*I* > 2σ(*I*)] reflections	327929, 33017, 27967	146764, 26559, 20132
*R*_int_	0.045	0.039
(sin θ/λ)_max_ (Å^−1^)	0.806	0.781

Refinement		
*R*[*F* ^2^ > 2σ(*F* ^2^)], *wR*(*F* ^2^), *S*	0.052, 0.130, 1.10	0.044, 0.118, 1.02
No. of reflections	33017	26559
No. of parameters	1028	979
H-atom treatment	H-atom parameters constrained	H-atom parameters constrained
Δρ_max_, Δρ_min_ (e Å^−3^)	1.00, −0.60	0.74, −0.96

Computer programs: *APEX3* and *SAINT* (Bruker, 2018 ▸), *SHELXS* (Sheldrick, 2015*a* ▸), *SHELXL* (Sheldrick, 2015*b* ▸) and *publCIF* (Westrip, 2010 ▸).

## supplementary crystallographic information

### Tris(dicyclohexylamido)(triphenylphosphine oxide)titanium methyltris(pentafluorophenyl)borate toluene sesquisolvate (1) Crystal data

**Table:**

[Ti(C_12_H_22_N)_3_(C_18_H_15_OP)](C_19_H_3_F_15_B)·1.5C_7_H_8_	*Z* = 2
*M**_r_* = 1532.31	*F*(000) = 1606
Triclinic, *P*1	*D*_x_ = 1.355 Mg m^−^^3^
*a* = 13.8643 (5) Å	Mo *K*α radiation, λ = 0.71073 Å
*b* = 14.3017 (5) Å	Cell parameters from 9161 reflections
*c* = 20.0502 (7) Å	θ = 2.4–34.2°
α = 86.4852 (13)°	µ = 0.22 mm^−^^1^
β = 80.0328 (13)°	*T* = 100 K
γ = 73.6493 (12)°	Block, yellow
*V* = 3756.9 (2) Å^3^	0.15 × 0.08 × 0.07 mm

### Tris(dicyclohexylamido)(triphenylphosphine oxide)titanium methyltris(pentafluorophenyl)borate toluene sesquisolvate (1) Data collection

**Table:**

Bruker Photon III CPAD diffractometer	27967 reflections with *I* > 2σ(*I*)
Radiation source: IµS microfocus	*R*_int_ = 0.045
φ and ω scans	θ_max_ = 35.0°, θ_min_ = 1.5°
Absorption correction: multi-scan (*SADABS*; Krause *et al.*, 2015)	*h* = −22→22
*T*_min_ = 0.958, *T*_max_ = 1.000	*k* = −23→23
327929 measured reflections	*l* = −32→32
33017 independent reflections	

### Tris(dicyclohexylamido)(triphenylphosphine oxide)titanium methyltris(pentafluorophenyl)borate toluene sesquisolvate (1) Refinement

**Table:**

Refinement on *F*^2^	Primary atom site location: structure-invariant direct methods
Least-squares matrix: full	Secondary atom site location: difference Fourier map
*R*[*F*^2^ > 2σ(*F*^2^)] = 0.052	Hydrogen site location: difference Fourier map
*wR*(*F*^2^) = 0.130	H-atom parameters constrained
*S* = 1.10	*w* = 1/[σ^2^(*F*_o_^2^) + (0.050*P*)^2^ + 2.*P*] where *P* = (*F*_o_^2^ + 2*F*_c_^2^)/3
33017 reflections	(Δ/σ)_max_ = 0.002
1028 parameters	Δρ_max_ = 1.00 e Å^−^^3^
0 restraints	Δρ_min_ = −0.60 e Å^−^^3^

### Tris(dicyclohexylamido)(triphenylphosphine oxide)titanium methyltris(pentafluorophenyl)borate toluene sesquisolvate (1) Special details

**Table:**

Geometry. All esds (except the esd in the dihedral angle between two l.s. planes) are estimated using the full covariance matrix. The cell esds are taken into account individually in the estimation of esds in distances, angles and torsion angles; correlations between esds in cell parameters are only used when they are defined by crystal symmetry. An approximate (isotropic) treatment of cell esds is used for estimating esds involving l.s. planes.

### Tris(dicyclohexylamido)(triphenylphosphine oxide)titanium methyltris(pentafluorophenyl)borate toluene sesquisolvate (1) Fractional atomic coordinates and isotropic or equivalent isotropic displacement parameters (Å^2^)

**Table:**

	*x*	*y*	*z*	*U*_iso_*/*U*_eq_	Occ. (<1)
Ti1	0.18373 (2)	0.73812 (2)	0.28951 (2)	0.01089 (3)	
P1	0.39386 (2)	0.83191 (2)	0.27325 (2)	0.01232 (5)	
O1	0.29851 (6)	0.79577 (6)	0.28627 (4)	0.01511 (13)	
N1	0.22191 (7)	0.63370 (6)	0.22745 (4)	0.01358 (14)	
N2	0.13961 (6)	0.69553 (6)	0.37850 (4)	0.01278 (14)	
N3	0.06758 (7)	0.83131 (6)	0.26314 (5)	0.01459 (15)	
C1	0.27392 (8)	0.66344 (8)	0.16157 (5)	0.01627 (17)	
H1	0.268489	0.734286	0.164834	0.020*	
C2	0.22418 (10)	0.65274 (10)	0.10092 (6)	0.0228 (2)	
H2A	0.224265	0.583917	0.097677	0.027*	
H2B	0.152446	0.693057	0.108088	0.027*	
C3	0.28018 (13)	0.68405 (12)	0.03472 (7)	0.0330 (3)	
H3A	0.246831	0.675068	−0.003333	0.040*	
H3B	0.276138	0.754076	0.036657	0.040*	
C4	0.39139 (13)	0.62480 (13)	0.02228 (7)	0.0346 (3)	
H4A	0.426382	0.646969	−0.020474	0.041*	
H4B	0.395683	0.555153	0.017705	0.041*	
C5	0.44403 (11)	0.63667 (11)	0.08093 (7)	0.0284 (3)	
H5A	0.446256	0.705116	0.082157	0.034*	
H5B	0.515049	0.594629	0.073452	0.034*	
C6	0.38837 (9)	0.60945 (9)	0.14907 (6)	0.01980 (19)	
H6A	0.420353	0.624747	0.185970	0.024*	
H6B	0.396545	0.538357	0.150534	0.024*	
C7	0.21749 (8)	0.53150 (7)	0.22859 (6)	0.01637 (17)	
H7	0.264945	0.498732	0.188091	0.020*	
C8	0.11123 (9)	0.52357 (8)	0.22391 (6)	0.01985 (19)	
H8A	0.090058	0.555784	0.181602	0.024*	
H8B	0.061981	0.557731	0.262533	0.024*	
C9	0.10943 (11)	0.41712 (10)	0.22469 (8)	0.0289 (3)	
H9A	0.039123	0.414366	0.223770	0.035*	
H9B	0.153540	0.384673	0.183626	0.035*	
C10	0.14631 (13)	0.36288 (10)	0.28744 (8)	0.0331 (3)	
H10A	0.148711	0.293324	0.284938	0.040*	
H10B	0.097784	0.390419	0.328355	0.040*	
C11	0.25238 (12)	0.37128 (9)	0.29298 (7)	0.0285 (3)	
H11A	0.272415	0.340246	0.335865	0.034*	
H11B	0.302498	0.336208	0.255056	0.034*	
C12	0.25404 (10)	0.47751 (8)	0.29113 (6)	0.0209 (2)	
H12A	0.210011	0.510412	0.332070	0.025*	
H12B	0.324353	0.480307	0.291971	0.025*	
C13	0.22782 (8)	0.66381 (7)	0.41487 (5)	0.01345 (16)	
H13	0.289184	0.654740	0.378798	0.016*	
C14	0.24348 (8)	0.56645 (8)	0.45425 (5)	0.01650 (17)	
H14A	0.190653	0.572927	0.495135	0.020*	
H14B	0.237338	0.515166	0.425556	0.020*	
C15	0.34950 (9)	0.53808 (9)	0.47484 (6)	0.0211 (2)	
H15A	0.360253	0.474906	0.499623	0.025*	
H15B	0.401881	0.529853	0.433634	0.025*	
C16	0.36197 (9)	0.61511 (10)	0.51958 (6)	0.0229 (2)	
H16A	0.432541	0.596693	0.529360	0.027*	
H16B	0.315126	0.617890	0.563153	0.027*	
C17	0.33926 (9)	0.71536 (9)	0.48515 (6)	0.0206 (2)	
H17A	0.392960	0.715551	0.445584	0.025*	
H17B	0.340164	0.764965	0.517195	0.025*	
C18	0.23552 (8)	0.74182 (8)	0.46184 (6)	0.01700 (18)	
H18A	0.224662	0.805365	0.437536	0.020*	
H18B	0.181264	0.748616	0.501919	0.020*	
C19	0.03521 (8)	0.69972 (8)	0.41294 (5)	0.01412 (16)	
H19	−0.011166	0.741660	0.383214	0.017*	
C20	0.01124 (8)	0.60065 (8)	0.41753 (6)	0.01755 (18)	
H20A	0.033415	0.568953	0.372809	0.021*	
H20B	0.049367	0.557822	0.450487	0.021*	
C21	−0.10304 (9)	0.61381 (9)	0.43976 (6)	0.0209 (2)	
H21A	−0.140678	0.652796	0.405106	0.025*	
H21B	−0.116775	0.549250	0.443456	0.025*	
C22	−0.14084 (9)	0.66486 (10)	0.50781 (6)	0.0245 (2)	
H22A	−0.110029	0.621966	0.543659	0.029*	
H22B	−0.215737	0.677482	0.519085	0.029*	
C23	−0.11296 (9)	0.76103 (10)	0.50566 (7)	0.0244 (2)	
H23A	−0.133469	0.790155	0.551253	0.029*	
H23B	−0.151090	0.806881	0.474083	0.029*	
C24	0.00140 (8)	0.74707 (8)	0.48285 (6)	0.01853 (18)	
H24A	0.039743	0.705173	0.516088	0.022*	
H24B	0.016393	0.811001	0.480693	0.022*	
C25A	0.02967 (11)	0.93444 (9)	0.28598 (7)	0.0167 (2)	0.872 (3)
H25A	−0.040809	0.960850	0.275167	0.020*	0.872 (3)
C26A	0.02251 (15)	0.94324 (11)	0.36217 (8)	0.0304 (4)	0.872 (3)
H26A	0.090977	0.916063	0.374829	0.036*	0.872 (3)
H26B	−0.022496	0.904829	0.386210	0.036*	0.872 (3)
C27A	−0.0198 (2)	1.05016 (14)	0.38418 (10)	0.0350 (5)	0.872 (3)
H27A	−0.091268	1.074648	0.376503	0.042*	0.872 (3)
H27B	−0.019407	1.053797	0.433283	0.042*	0.872 (3)
C28A	0.04142 (18)	1.11465 (12)	0.34602 (11)	0.0373 (4)	0.872 (3)
H28A	0.110597	1.095878	0.358230	0.045*	0.872 (3)
H28B	0.008383	1.183299	0.359195	0.045*	0.872 (3)
C29A	0.04929 (14)	1.10559 (11)	0.27040 (10)	0.0311 (4)	0.872 (3)
H29A	0.092797	1.145551	0.246529	0.037*	0.872 (3)
H29B	−0.019301	1.130965	0.257629	0.037*	0.872 (3)
C30A	0.09460 (15)	0.99918 (14)	0.24809 (14)	0.0224 (3)	0.872 (3)
H30A	0.165164	0.975131	0.257698	0.027*	0.872 (3)
H30B	0.096964	0.995259	0.198687	0.027*	0.872 (3)
C25B	0.0604 (9)	0.9168 (8)	0.3028 (6)	0.0205 (18)*	0.128 (3)
H25B	0.105441	0.893450	0.337856	0.025*	0.128 (3)
C26B	−0.0456 (10)	0.9620 (10)	0.3405 (7)	0.035 (3)*	0.128 (3)
H26C	−0.070380	0.910254	0.367030	0.042*	0.128 (3)
H26D	−0.091527	0.988301	0.307015	0.042*	0.128 (3)
C27B	−0.0524 (12)	1.0439 (14)	0.3885 (10)	0.033 (4)*	0.128 (3)
H27C	−0.012487	1.018548	0.425409	0.040*	0.128 (3)
H27D	−0.124075	1.074429	0.408735	0.040*	0.128 (3)
C28B	−0.0055 (15)	1.1207 (13)	0.3417 (9)	0.047 (4)*	0.128 (3)
H28C	−0.051836	1.150061	0.308917	0.057*	0.128 (3)
H28D	−0.003997	1.173647	0.370680	0.057*	0.128 (3)
C29B	0.0984 (10)	1.0802 (10)	0.3034 (7)	0.037 (3)*	0.128 (3)
H29C	0.148550	1.060042	0.334833	0.044*	0.128 (3)
H29D	0.117986	1.129536	0.271274	0.044*	0.128 (3)
C30B	0.0950 (14)	0.9918 (13)	0.2651 (8)	0.024 (4)*	0.128 (3)
H30C	0.050790	1.016111	0.230332	0.028*	0.128 (3)
H30D	0.164545	0.962043	0.240791	0.028*	0.128 (3)
C31	0.00393 (8)	0.80786 (8)	0.21756 (6)	0.01763 (18)	
H31	0.039993	0.740556	0.200795	0.021*	
C32	−0.10107 (9)	0.80337 (9)	0.25524 (6)	0.0216 (2)	
H32A	−0.092285	0.757197	0.294026	0.026*	
H32B	−0.140057	0.868514	0.273205	0.026*	
C33	−0.16072 (11)	0.77042 (12)	0.20778 (8)	0.0322 (3)	
H33A	−0.125158	0.702644	0.193564	0.039*	
H33B	−0.229385	0.771453	0.232384	0.039*	
C34	−0.17091 (12)	0.83629 (13)	0.14553 (8)	0.0364 (3)	
H34A	−0.206840	0.811739	0.114880	0.044*	
H34B	−0.211898	0.902895	0.159342	0.044*	
C35	−0.06702 (11)	0.83962 (12)	0.10843 (7)	0.0312 (3)	
H35A	−0.075482	0.884664	0.069022	0.037*	
H35B	−0.028422	0.773940	0.091256	0.037*	
C36	−0.00646 (10)	0.87351 (10)	0.15456 (6)	0.0245 (2)	
H36A	0.062078	0.871848	0.129452	0.029*	
H36B	−0.041611	0.941547	0.168353	0.029*	
C37	0.43656 (8)	0.84548 (8)	0.18492 (5)	0.01613 (17)	
C38	0.36575 (10)	0.89837 (9)	0.14514 (6)	0.0222 (2)	
H38	0.295837	0.921024	0.164381	0.027*	
C39	0.39753 (12)	0.91783 (10)	0.07746 (6)	0.0283 (3)	
H39	0.349574	0.954707	0.050574	0.034*	
C40	0.49955 (12)	0.88330 (10)	0.04912 (6)	0.0283 (3)	
H40	0.521204	0.896258	0.002747	0.034*	
C41	0.56970 (11)	0.83010 (11)	0.08824 (7)	0.0270 (2)	
H41	0.639241	0.806355	0.068416	0.032*	
C42	0.53928 (9)	0.81102 (9)	0.15645 (6)	0.0212 (2)	
H42	0.587761	0.775027	0.183283	0.025*	
C43	0.49374 (8)	0.75095 (7)	0.31144 (5)	0.01471 (16)	
C44	0.50403 (8)	0.65073 (8)	0.31449 (6)	0.01741 (18)	
H44	0.459325	0.625060	0.294935	0.021*	
C45	0.57986 (9)	0.58876 (9)	0.34621 (6)	0.0217 (2)	
H45	0.586608	0.520723	0.348843	0.026*	
C46	0.64578 (9)	0.62661 (10)	0.37407 (6)	0.0235 (2)	
H46	0.697564	0.584098	0.395687	0.028*	
C47	0.63673 (9)	0.72583 (10)	0.37063 (7)	0.0240 (2)	
H47	0.682418	0.750974	0.389543	0.029*	
C48	0.56067 (9)	0.78836 (9)	0.33944 (6)	0.01974 (19)	
H48	0.554115	0.856366	0.337134	0.024*	
C49	0.36942 (8)	0.94788 (7)	0.31135 (5)	0.01411 (16)	
C50	0.33452 (10)	0.95522 (8)	0.38112 (6)	0.0202 (2)	
H50	0.322453	0.900095	0.406409	0.024*	
C51	0.31750 (10)	1.04274 (8)	0.41345 (6)	0.0210 (2)	
H51	0.293285	1.047817	0.460765	0.025*	
C52	0.33600 (9)	1.12289 (8)	0.37640 (7)	0.0211 (2)	
H52	0.323169	1.183218	0.398336	0.025*	
C53	0.37304 (11)	1.11543 (9)	0.30764 (7)	0.0251 (2)	
H53	0.386772	1.170282	0.282831	0.030*	
C54	0.39024 (9)	1.02796 (8)	0.27472 (6)	0.0202 (2)	
H54	0.415988	1.022838	0.227595	0.024*	
F1	0.86289 (8)	0.23884 (8)	0.15471 (6)	0.0434 (2)	
F2	0.93523 (10)	0.13184 (10)	0.04461 (7)	0.0578 (3)	
F3	0.82569 (11)	0.02381 (8)	0.00024 (5)	0.0538 (3)	
F4	0.64270 (11)	0.01982 (8)	0.07450 (5)	0.0518 (3)	
F5	0.56753 (9)	0.12686 (8)	0.18431 (5)	0.0424 (2)	
F6	0.72872 (8)	0.06269 (7)	0.28033 (5)	0.0397 (2)	
F7	0.66236 (10)	−0.01679 (7)	0.39566 (6)	0.0467 (3)	
F8	0.51309 (9)	0.09344 (8)	0.48955 (5)	0.0409 (2)	
F9	0.43106 (7)	0.28459 (7)	0.46298 (4)	0.03347 (19)	
F10	0.49707 (7)	0.36744 (6)	0.34873 (4)	0.02749 (16)	
F11	0.78875 (7)	0.25151 (7)	0.33386 (5)	0.03436 (19)	
F12	0.88489 (7)	0.37892 (8)	0.35793 (5)	0.0385 (2)	
F13	0.86846 (7)	0.54676 (7)	0.28376 (5)	0.0348 (2)	
F14	0.75070 (9)	0.58404 (7)	0.18391 (6)	0.0439 (2)	
F15	0.65663 (9)	0.45785 (7)	0.15716 (5)	0.0412 (2)	
C55	0.70693 (11)	0.19516 (9)	0.17360 (7)	0.0255 (2)	
C56	0.80222 (12)	0.18848 (10)	0.13668 (8)	0.0301 (3)	
C57	0.84316 (13)	0.13138 (11)	0.07902 (8)	0.0360 (3)	
C58	0.78821 (16)	0.07678 (11)	0.05702 (8)	0.0388 (4)	
C59	0.69526 (15)	0.07655 (11)	0.09369 (7)	0.0362 (3)	
C60	0.65791 (13)	0.13375 (10)	0.15005 (7)	0.0311 (3)	
C61	0.61327 (10)	0.21929 (9)	0.30580 (6)	0.0215 (2)	
C62	0.65280 (11)	0.12165 (10)	0.32273 (7)	0.0272 (2)	
C63	0.62040 (13)	0.07831 (10)	0.38313 (8)	0.0302 (3)	
C64	0.54540 (12)	0.13302 (10)	0.43069 (7)	0.0279 (3)	
C65	0.50426 (10)	0.23019 (10)	0.41692 (6)	0.0242 (2)	
C66	0.53907 (10)	0.27061 (9)	0.35634 (6)	0.0215 (2)	
C67	0.71135 (9)	0.34890 (9)	0.24661 (6)	0.0213 (2)	
C68	0.77432 (9)	0.33369 (9)	0.29563 (7)	0.0233 (2)	
C69	0.82748 (10)	0.39763 (10)	0.30847 (7)	0.0260 (2)	
C70	0.82006 (10)	0.48246 (10)	0.27078 (7)	0.0265 (2)	
C71	0.76040 (11)	0.50096 (10)	0.22072 (8)	0.0285 (3)	
C72	0.70896 (11)	0.43481 (10)	0.20934 (7)	0.0261 (2)	
C73	0.53849 (11)	0.33612 (11)	0.20648 (8)	0.0295 (3)	
H73A	0.488147	0.298778	0.216137	0.044*	
H73B	0.552898	0.347609	0.157545	0.044*	
H73C	0.511496	0.398750	0.229659	0.044*	
B1	0.64436 (11)	0.27427 (10)	0.23356 (7)	0.0219 (2)	
C74A	0.21455 (17)	0.22739 (16)	0.07877 (11)	0.0353 (5)	0.764 (4)
C75A	0.2932 (2)	0.19832 (19)	0.11473 (13)	0.0451 (7)	0.764 (4)
H75A	0.299020	0.140843	0.141923	0.054*	0.764 (4)
C76A	0.3634 (2)	0.2484 (3)	0.11309 (18)	0.0588 (9)	0.764 (4)
H76A	0.417270	0.224698	0.138591	0.071*	0.764 (4)
C77A	0.3585 (2)	0.3297 (3)	0.07669 (18)	0.0642 (10)	0.764 (4)
H77A	0.407783	0.364145	0.077302	0.077*	0.764 (4)
C78A	0.2859 (2)	0.36319 (19)	0.03966 (14)	0.0496 (7)	0.764 (4)
H78A	0.284443	0.420900	0.013261	0.060*	0.764 (4)
C79A	0.20963 (17)	0.31452 (19)	0.03848 (12)	0.0410 (6)	0.764 (4)
H79A	0.157233	0.338941	0.011879	0.049*	0.764 (4)
C80A	0.1332 (3)	0.1767 (3)	0.0810 (2)	0.0693 (10)	0.764 (4)
H80A	0.070158	0.214526	0.108354	0.104*	0.764 (4)
H80B	0.154952	0.111645	0.101295	0.104*	0.764 (4)
H80C	0.121125	0.170821	0.034934	0.104*	0.764 (4)
C74B	0.2613 (5)	0.3159 (4)	0.0373 (3)	0.058 (3)*	0.236 (4)
C75B	0.1846 (4)	0.2695 (4)	0.0431 (3)	0.0323 (14)*	0.236 (4)
H75B	0.133210	0.289779	0.015325	0.039*	0.236 (4)
C76B	0.1829 (5)	0.1935 (5)	0.0896 (3)	0.058 (2)*	0.236 (4)
H76B	0.130391	0.161833	0.093526	0.069*	0.236 (4)
C77B	0.2580 (6)	0.1639 (5)	0.1302 (4)	0.076 (4)*	0.236 (4)
H77B	0.256854	0.111939	0.161978	0.092*	0.236 (4)
C78B	0.3348 (5)	0.2102 (6)	0.1244 (3)	0.056 (3)*	0.236 (4)
H78B	0.386138	0.189990	0.152230	0.067*	0.236 (4)
C79B	0.3365 (4)	0.2862 (5)	0.0780 (3)	0.0451 (19)*	0.236 (4)
H79B	0.388960	0.317936	0.074029	0.054*	0.236 (4)
C80B	0.2585 (15)	0.3877 (13)	−0.0088 (10)	0.107 (6)*	0.236 (4)
H80D	0.298969	0.361083	−0.051955	0.161*	0.236 (4)
H80E	0.286539	0.436674	0.006674	0.161*	0.236 (4)
H80F	0.187763	0.418033	−0.014998	0.161*	0.236 (4)
C81	0.0349 (3)	0.5322 (3)	−0.02186 (17)	0.0435 (8)	0.5
C82	0.0012 (9)	0.4594 (9)	−0.0452 (6)	0.075 (4)	0.5
H82	0.014630	0.445728	−0.092175	0.090*	0.5
C83	−0.0526 (3)	0.4059 (4)	0.0003 (3)	0.0705 (17)	0.5
H83	−0.076604	0.357109	−0.016171	0.085*	0.5
C84	−0.0708 (5)	0.4239 (7)	0.0686 (5)	0.063 (2)	0.5
H84	−0.107429	0.387985	0.099390	0.076*	0.5
C85	−0.0351 (3)	0.4949 (3)	0.09234 (18)	0.0407 (7)	0.5
H85	−0.045349	0.506993	0.139465	0.049*	0.5
C86	0.0149 (6)	0.5471 (5)	0.0468 (3)	0.0301 (9)	0.5
H86	0.037228	0.596802	0.063584	0.036*	0.5
C87	0.0926 (8)	0.5923 (8)	−0.0683 (4)	0.097 (4)	0.5
H87A	0.080325	0.656210	−0.048311	0.146*	0.5
H87B	0.069269	0.600720	−0.112305	0.146*	0.5
H87C	0.165601	0.558829	−0.074512	0.146*	0.5

### Tris(dicyclohexylamido)(triphenylphosphine oxide)titanium methyltris(pentafluorophenyl)borate toluene sesquisolvate (1) Atomic displacement parameters (Å^2^)

**Table:**

	*U*^11^	*U*^22^	*U*^33^	*U*^12^	*U*^13^	*U*^23^
Ti1	0.01189 (7)	0.01054 (7)	0.01084 (7)	−0.00382 (5)	−0.00240 (5)	0.00025 (5)
P1	0.01357 (10)	0.01121 (10)	0.01317 (10)	−0.00491 (8)	−0.00254 (8)	0.00043 (8)
O1	0.0156 (3)	0.0150 (3)	0.0169 (3)	−0.0073 (3)	−0.0036 (3)	0.0008 (3)
N1	0.0161 (4)	0.0110 (3)	0.0143 (3)	−0.0046 (3)	−0.0027 (3)	0.0001 (3)
N2	0.0127 (3)	0.0141 (3)	0.0121 (3)	−0.0042 (3)	−0.0028 (3)	0.0007 (3)
N3	0.0162 (4)	0.0127 (3)	0.0153 (4)	−0.0034 (3)	−0.0047 (3)	−0.0006 (3)
C1	0.0206 (4)	0.0146 (4)	0.0136 (4)	−0.0047 (3)	−0.0026 (3)	−0.0007 (3)
C2	0.0262 (5)	0.0260 (5)	0.0161 (4)	−0.0053 (4)	−0.0060 (4)	−0.0003 (4)
C3	0.0467 (8)	0.0370 (7)	0.0159 (5)	−0.0133 (6)	−0.0057 (5)	0.0045 (5)
C4	0.0439 (8)	0.0446 (8)	0.0177 (5)	−0.0215 (7)	0.0063 (5)	−0.0070 (5)
C5	0.0314 (6)	0.0341 (7)	0.0219 (5)	−0.0179 (5)	0.0064 (5)	−0.0060 (5)
C6	0.0192 (5)	0.0217 (5)	0.0186 (5)	−0.0075 (4)	0.0004 (4)	−0.0029 (4)
C7	0.0182 (4)	0.0128 (4)	0.0181 (4)	−0.0049 (3)	−0.0015 (3)	−0.0015 (3)
C8	0.0195 (5)	0.0199 (5)	0.0218 (5)	−0.0091 (4)	0.0003 (4)	−0.0058 (4)
C9	0.0303 (6)	0.0244 (6)	0.0349 (7)	−0.0158 (5)	0.0044 (5)	−0.0106 (5)
C10	0.0432 (8)	0.0188 (5)	0.0364 (7)	−0.0161 (5)	0.0098 (6)	−0.0042 (5)
C11	0.0390 (7)	0.0120 (4)	0.0293 (6)	−0.0040 (4)	0.0028 (5)	0.0018 (4)
C12	0.0267 (5)	0.0137 (4)	0.0210 (5)	−0.0032 (4)	−0.0047 (4)	0.0012 (4)
C13	0.0133 (4)	0.0142 (4)	0.0131 (4)	−0.0040 (3)	−0.0030 (3)	0.0015 (3)
C14	0.0171 (4)	0.0149 (4)	0.0172 (4)	−0.0044 (3)	−0.0033 (3)	0.0029 (3)
C15	0.0181 (5)	0.0202 (5)	0.0227 (5)	−0.0016 (4)	−0.0055 (4)	0.0059 (4)
C16	0.0201 (5)	0.0299 (6)	0.0202 (5)	−0.0067 (4)	−0.0099 (4)	0.0066 (4)
C17	0.0193 (5)	0.0259 (5)	0.0202 (5)	−0.0091 (4)	−0.0084 (4)	0.0020 (4)
C18	0.0178 (4)	0.0166 (4)	0.0185 (4)	−0.0055 (3)	−0.0067 (3)	−0.0002 (3)
C19	0.0128 (4)	0.0163 (4)	0.0132 (4)	−0.0041 (3)	−0.0017 (3)	−0.0005 (3)
C20	0.0158 (4)	0.0191 (4)	0.0184 (4)	−0.0074 (3)	0.0010 (3)	−0.0027 (3)
C21	0.0174 (5)	0.0269 (5)	0.0203 (5)	−0.0106 (4)	−0.0008 (4)	0.0000 (4)
C22	0.0176 (5)	0.0323 (6)	0.0223 (5)	−0.0089 (4)	0.0048 (4)	−0.0034 (4)
C23	0.0163 (5)	0.0273 (6)	0.0262 (5)	−0.0028 (4)	0.0034 (4)	−0.0089 (4)
C24	0.0156 (4)	0.0205 (5)	0.0182 (4)	−0.0036 (4)	0.0000 (3)	−0.0056 (4)
C25A	0.0192 (5)	0.0119 (5)	0.0184 (5)	−0.0016 (4)	−0.0063 (5)	−0.0008 (4)
C26A	0.0517 (10)	0.0154 (6)	0.0206 (6)	0.0002 (6)	−0.0111 (6)	−0.0031 (5)
C27A	0.0556 (16)	0.0174 (7)	0.0258 (8)	0.0024 (9)	−0.0080 (9)	−0.0078 (5)
C28A	0.0478 (11)	0.0168 (6)	0.0497 (11)	−0.0010 (6)	−0.0242 (9)	−0.0113 (6)
C29A	0.0356 (8)	0.0138 (6)	0.0445 (9)	−0.0069 (5)	−0.0077 (7)	−0.0013 (6)
C30A	0.0237 (7)	0.0135 (6)	0.0298 (10)	−0.0064 (5)	−0.0018 (8)	−0.0006 (7)
C31	0.0183 (4)	0.0177 (4)	0.0171 (4)	−0.0030 (3)	−0.0071 (3)	0.0006 (3)
C32	0.0199 (5)	0.0243 (5)	0.0233 (5)	−0.0086 (4)	−0.0075 (4)	0.0027 (4)
C33	0.0275 (6)	0.0350 (7)	0.0406 (8)	−0.0124 (5)	−0.0174 (6)	0.0009 (6)
C34	0.0298 (7)	0.0467 (9)	0.0347 (7)	−0.0052 (6)	−0.0197 (6)	−0.0011 (6)
C35	0.0316 (6)	0.0383 (7)	0.0213 (5)	−0.0002 (5)	−0.0135 (5)	−0.0016 (5)
C36	0.0263 (6)	0.0268 (6)	0.0192 (5)	−0.0042 (4)	−0.0069 (4)	0.0041 (4)
C37	0.0205 (4)	0.0151 (4)	0.0142 (4)	−0.0077 (3)	−0.0019 (3)	−0.0001 (3)
C38	0.0267 (5)	0.0231 (5)	0.0162 (4)	−0.0057 (4)	−0.0042 (4)	0.0015 (4)
C39	0.0416 (7)	0.0269 (6)	0.0168 (5)	−0.0098 (5)	−0.0072 (5)	0.0045 (4)
C40	0.0436 (7)	0.0289 (6)	0.0163 (5)	−0.0199 (6)	0.0009 (5)	0.0007 (4)
C41	0.0278 (6)	0.0337 (6)	0.0217 (5)	−0.0169 (5)	0.0058 (4)	−0.0048 (5)
C42	0.0204 (5)	0.0251 (5)	0.0188 (5)	−0.0094 (4)	0.0008 (4)	−0.0019 (4)
C43	0.0143 (4)	0.0145 (4)	0.0151 (4)	−0.0037 (3)	−0.0022 (3)	0.0008 (3)
C44	0.0172 (4)	0.0146 (4)	0.0190 (4)	−0.0034 (3)	−0.0012 (3)	0.0007 (3)
C45	0.0207 (5)	0.0176 (5)	0.0222 (5)	−0.0001 (4)	−0.0006 (4)	0.0033 (4)
C46	0.0186 (5)	0.0265 (5)	0.0198 (5)	0.0016 (4)	−0.0028 (4)	0.0035 (4)
C47	0.0187 (5)	0.0287 (6)	0.0246 (5)	−0.0035 (4)	−0.0082 (4)	−0.0009 (4)
C48	0.0176 (4)	0.0201 (5)	0.0224 (5)	−0.0049 (4)	−0.0059 (4)	−0.0002 (4)
C49	0.0152 (4)	0.0119 (4)	0.0161 (4)	−0.0049 (3)	−0.0029 (3)	−0.0003 (3)
C50	0.0288 (5)	0.0150 (4)	0.0170 (4)	−0.0078 (4)	−0.0014 (4)	−0.0006 (3)
C51	0.0254 (5)	0.0168 (4)	0.0203 (5)	−0.0051 (4)	−0.0022 (4)	−0.0039 (4)
C52	0.0196 (5)	0.0140 (4)	0.0296 (6)	−0.0041 (4)	−0.0029 (4)	−0.0056 (4)
C53	0.0311 (6)	0.0131 (4)	0.0306 (6)	−0.0087 (4)	0.0014 (5)	−0.0006 (4)
C54	0.0256 (5)	0.0144 (4)	0.0206 (5)	−0.0078 (4)	−0.0003 (4)	0.0008 (4)
F1	0.0294 (4)	0.0436 (5)	0.0540 (6)	−0.0099 (4)	0.0070 (4)	−0.0175 (5)
F2	0.0496 (7)	0.0592 (7)	0.0499 (7)	−0.0031 (6)	0.0169 (5)	−0.0156 (6)
F3	0.0899 (9)	0.0366 (5)	0.0244 (4)	0.0016 (6)	−0.0101 (5)	−0.0082 (4)
F4	0.0992 (10)	0.0384 (5)	0.0334 (5)	−0.0354 (6)	−0.0257 (6)	0.0017 (4)
F5	0.0563 (6)	0.0450 (6)	0.0386 (5)	−0.0342 (5)	−0.0080 (4)	0.0003 (4)
F6	0.0490 (6)	0.0244 (4)	0.0349 (5)	0.0045 (4)	−0.0031 (4)	0.0021 (3)
F7	0.0743 (8)	0.0222 (4)	0.0421 (6)	−0.0093 (4)	−0.0164 (5)	0.0117 (4)
F8	0.0640 (7)	0.0418 (5)	0.0267 (4)	−0.0320 (5)	−0.0088 (4)	0.0119 (4)
F9	0.0365 (5)	0.0404 (5)	0.0252 (4)	−0.0168 (4)	0.0020 (3)	−0.0046 (3)
F10	0.0332 (4)	0.0202 (3)	0.0278 (4)	−0.0051 (3)	−0.0051 (3)	−0.0008 (3)
F11	0.0328 (4)	0.0287 (4)	0.0464 (5)	−0.0119 (3)	−0.0181 (4)	0.0128 (4)
F12	0.0303 (4)	0.0460 (5)	0.0464 (5)	−0.0163 (4)	−0.0164 (4)	0.0016 (4)
F13	0.0282 (4)	0.0326 (4)	0.0471 (5)	−0.0170 (3)	0.0030 (4)	−0.0113 (4)
F14	0.0581 (6)	0.0289 (4)	0.0522 (6)	−0.0248 (4)	−0.0118 (5)	0.0118 (4)
F15	0.0587 (6)	0.0387 (5)	0.0378 (5)	−0.0269 (5)	−0.0225 (5)	0.0169 (4)
C55	0.0354 (6)	0.0192 (5)	0.0223 (5)	−0.0080 (5)	−0.0050 (5)	0.0012 (4)
C56	0.0357 (7)	0.0229 (6)	0.0285 (6)	−0.0046 (5)	−0.0012 (5)	−0.0019 (5)
C57	0.0428 (8)	0.0286 (7)	0.0278 (6)	−0.0001 (6)	0.0024 (6)	−0.0019 (5)
C58	0.0673 (11)	0.0221 (6)	0.0218 (6)	−0.0016 (6)	−0.0111 (6)	−0.0003 (5)
C59	0.0672 (11)	0.0236 (6)	0.0235 (6)	−0.0173 (6)	−0.0158 (6)	0.0038 (5)
C60	0.0486 (8)	0.0249 (6)	0.0240 (6)	−0.0162 (6)	−0.0086 (6)	0.0035 (5)
C61	0.0247 (5)	0.0190 (5)	0.0229 (5)	−0.0088 (4)	−0.0060 (4)	0.0014 (4)
C62	0.0350 (7)	0.0201 (5)	0.0265 (6)	−0.0067 (5)	−0.0078 (5)	0.0016 (4)
C63	0.0444 (8)	0.0204 (5)	0.0303 (6)	−0.0127 (5)	−0.0145 (6)	0.0067 (5)
C64	0.0400 (7)	0.0296 (6)	0.0227 (5)	−0.0217 (5)	−0.0104 (5)	0.0063 (5)
C65	0.0281 (6)	0.0283 (6)	0.0218 (5)	−0.0158 (5)	−0.0056 (4)	−0.0009 (4)
C66	0.0254 (5)	0.0197 (5)	0.0232 (5)	−0.0105 (4)	−0.0073 (4)	0.0015 (4)
C67	0.0225 (5)	0.0176 (5)	0.0232 (5)	−0.0056 (4)	−0.0012 (4)	−0.0015 (4)
C68	0.0205 (5)	0.0204 (5)	0.0284 (6)	−0.0056 (4)	−0.0029 (4)	0.0003 (4)
C69	0.0182 (5)	0.0290 (6)	0.0307 (6)	−0.0068 (4)	−0.0020 (4)	−0.0044 (5)
C70	0.0217 (5)	0.0243 (5)	0.0333 (6)	−0.0102 (4)	0.0049 (5)	−0.0091 (5)
C71	0.0326 (6)	0.0207 (5)	0.0323 (6)	−0.0117 (5)	0.0015 (5)	−0.0005 (5)
C72	0.0313 (6)	0.0221 (5)	0.0261 (6)	−0.0100 (5)	−0.0042 (5)	0.0012 (4)
C73	0.0295 (6)	0.0300 (6)	0.0303 (6)	−0.0095 (5)	−0.0086 (5)	0.0068 (5)
B1	0.0255 (6)	0.0188 (5)	0.0223 (6)	−0.0076 (4)	−0.0045 (5)	0.0015 (4)
C74A	0.0352 (10)	0.0353 (10)	0.0365 (10)	−0.0106 (8)	−0.0021 (8)	−0.0160 (8)
C75A	0.0489 (14)	0.0408 (12)	0.0375 (11)	0.0099 (11)	−0.0176 (10)	−0.0136 (9)
C76A	0.0378 (13)	0.071 (2)	0.0635 (18)	0.0009 (13)	−0.0118 (12)	−0.0377 (16)
C77A	0.0330 (12)	0.089 (2)	0.072 (2)	−0.0218 (14)	0.0105 (12)	−0.0474 (19)
C78A	0.0618 (16)	0.0395 (12)	0.0435 (13)	−0.0190 (11)	0.0158 (12)	−0.0151 (10)
C79A	0.0286 (10)	0.0505 (14)	0.0369 (11)	0.0029 (9)	−0.0057 (8)	−0.0115 (9)
C80A	0.071 (2)	0.076 (2)	0.077 (2)	−0.0451 (18)	−0.0034 (17)	−0.0278 (18)
C81	0.0376 (16)	0.053 (2)	0.0275 (14)	0.0069 (15)	−0.0051 (12)	−0.0004 (13)
C82	0.049 (5)	0.105 (7)	0.060 (4)	0.020 (4)	−0.028 (4)	−0.046 (4)
C83	0.0343 (19)	0.078 (3)	0.104 (4)	−0.011 (2)	−0.013 (2)	−0.057 (3)
C84	0.0329 (19)	0.061 (4)	0.094 (5)	−0.018 (2)	0.012 (2)	−0.021 (3)
C85	0.0348 (16)	0.057 (2)	0.0302 (15)	−0.0152 (15)	0.0014 (12)	−0.0076 (14)
C86	0.028 (2)	0.0357 (19)	0.0223 (18)	−0.0005 (15)	−0.0045 (14)	−0.0045 (14)
C87	0.126 (9)	0.085 (6)	0.044 (3)	0.005 (5)	0.016 (4)	0.032 (4)

### Tris(dicyclohexylamido)(triphenylphosphine oxide)titanium methyltris(pentafluorophenyl)borate toluene sesquisolvate (1) Geometric parameters (Å, º)

**Table:**

Ti1—N1	1.9009 (9)	C34—C35	1.514 (2)
Ti1—N2	1.9047 (9)	C34—H34A	0.9900
Ti1—N3	1.9102 (9)	C34—H34B	0.9900
Ti1—O1	1.9782 (8)	C35—C36	1.5324 (19)
Ti1—C25B	2.644 (11)	C35—H35A	0.9900
P1—O1	1.5269 (8)	C35—H35B	0.9900
P1—C37	1.7845 (11)	C36—H36A	0.9900
P1—C49	1.7884 (10)	C36—H36B	0.9900
P1—C43	1.7889 (11)	C37—C38	1.3968 (16)
N1—C7	1.4786 (13)	C37—C42	1.3981 (16)
N1—C1	1.4898 (14)	C38—C39	1.3888 (17)
N2—C19	1.4784 (13)	C38—H38	0.9500
N2—C13	1.4794 (13)	C39—C40	1.389 (2)
N3—C25B	1.470 (10)	C39—H39	0.9500
N3—C31	1.4866 (14)	C40—C41	1.384 (2)
N3—C25A	1.4914 (15)	C40—H40	0.9500
C1—C2	1.5331 (16)	C41—C42	1.3936 (17)
C1—C6	1.5405 (16)	C41—H41	0.9500
C1—H1	1.0000	C42—H42	0.9500
C2—C3	1.5267 (19)	C43—C44	1.3984 (15)
C2—H2A	0.9900	C43—C48	1.3998 (15)
C2—H2B	0.9900	C44—C45	1.3904 (16)
C3—C4	1.521 (2)	C44—H44	0.9500
C3—H3A	0.9900	C45—C46	1.3903 (19)
C3—H3B	0.9900	C45—H45	0.9500
C4—C5	1.528 (2)	C46—C47	1.3874 (19)
C4—H4A	0.9900	C46—H46	0.9500
C4—H4B	0.9900	C47—C48	1.3897 (16)
C5—C6	1.5336 (17)	C47—H47	0.9500
C5—H5A	0.9900	C48—H48	0.9500
C5—H5B	0.9900	C49—C54	1.3942 (15)
C6—H6A	0.9900	C49—C50	1.3989 (15)
C6—H6B	0.9900	C50—C51	1.3867 (16)
C7—C12	1.5186 (16)	C50—H50	0.9500
C7—C8	1.5283 (16)	C51—C52	1.3881 (17)
C7—H7	1.0000	C51—H51	0.9500
C8—C9	1.5286 (17)	C52—C53	1.3849 (19)
C8—H8A	0.9900	C52—H52	0.9500
C8—H8B	0.9900	C53—C54	1.3916 (17)
C9—C10	1.525 (2)	C53—H53	0.9500
C9—H9A	0.9900	C54—H54	0.9500
C9—H9B	0.9900	F1—C56	1.3548 (19)
C10—C11	1.532 (2)	F2—C57	1.343 (2)
C10—H10A	0.9900	F3—C58	1.3488 (18)
C10—H10B	0.9900	F4—C59	1.3436 (19)
C11—C12	1.5239 (17)	F5—C60	1.3473 (19)
C11—H11A	0.9900	F6—C62	1.3490 (17)
C11—H11B	0.9900	F7—C63	1.3470 (16)
C12—H12A	0.9900	F8—C64	1.3386 (15)
C12—H12B	0.9900	F9—C65	1.3442 (16)
C13—C14	1.5376 (14)	F10—C66	1.3531 (14)
C13—C18	1.5407 (15)	F11—C68	1.3488 (15)
C13—H13	1.0000	F12—C69	1.3451 (17)
C14—C15	1.5327 (16)	F13—C70	1.3420 (15)
C14—H14A	0.9900	F14—C71	1.3458 (16)
C14—H14B	0.9900	F15—C72	1.3476 (17)
C15—C16	1.5265 (19)	C55—C56	1.379 (2)
C15—H15A	0.9900	C55—C60	1.398 (2)
C15—H15B	0.9900	C55—B1	1.6526 (19)
C16—C17	1.5267 (17)	C56—C57	1.398 (2)
C16—H16A	0.9900	C57—C58	1.371 (3)
C16—H16B	0.9900	C58—C59	1.369 (3)
C17—C18	1.5280 (15)	C59—C60	1.374 (2)
C17—H17A	0.9900	C61—C66	1.3887 (18)
C17—H17B	0.9900	C61—C62	1.3932 (17)
C18—H18A	0.9900	C61—B1	1.6545 (18)
C18—H18B	0.9900	C62—C63	1.389 (2)
C19—C24	1.5340 (15)	C63—C64	1.376 (2)
C19—C20	1.5379 (15)	C64—C65	1.376 (2)
C19—H19	1.0000	C65—C66	1.3823 (18)
C20—C21	1.5305 (15)	C67—C68	1.3925 (18)
C20—H20A	0.9900	C67—C72	1.3936 (17)
C20—H20B	0.9900	C67—B1	1.6573 (18)
C21—C22	1.5260 (17)	C68—C69	1.3856 (18)
C21—H21A	0.9900	C69—C70	1.377 (2)
C21—H21B	0.9900	C70—C71	1.376 (2)
C22—C23	1.5262 (19)	C71—C72	1.3849 (19)
C22—H22A	0.9900	C73—B1	1.649 (2)
C22—H22B	0.9900	C73—H73A	0.9800
C23—C24	1.5313 (16)	C73—H73B	0.9800
C23—H23A	0.9900	C73—H73C	0.9800
C23—H23B	0.9900	C74A—C75A	1.364 (3)
C24—H24A	0.9900	C74A—C79A	1.435 (4)
C24—H24B	0.9900	C74A—C80A	1.496 (4)
C25A—C26A	1.524 (2)	C75A—C76A	1.356 (5)
C25A—C30A	1.546 (2)	C75A—H75A	0.9500
C25A—H25A	1.0000	C76A—C77A	1.326 (5)
C26A—C27A	1.538 (2)	C76A—H76A	0.9500
C26A—H26A	0.9900	C77A—C78A	1.318 (5)
C26A—H26B	0.9900	C77A—H77A	0.9500
C27A—C28A	1.512 (4)	C78A—C79A	1.425 (4)
C27A—H27A	0.9900	C78A—H78A	0.9500
C27A—H27B	0.9900	C79A—H79A	0.9500
C28A—C29A	1.512 (3)	C80A—H80A	0.9800
C28A—H28A	0.9900	C80A—H80B	0.9800
C28A—H28B	0.9900	C80A—H80C	0.9800
C29A—C30A	1.536 (2)	C74B—C80B	1.336 (19)
C29A—H29A	0.9900	C74B—C75B	1.3900
C29A—H29B	0.9900	C74B—C79B	1.3900
C30A—H30A	0.9900	C75B—C76B	1.3900
C30A—H30B	0.9900	C75B—H75B	0.9500
C25B—C30B	1.420 (19)	C76B—C77B	1.3900
C25B—C26B	1.513 (17)	C76B—H76B	0.9500
C25B—H25B	1.0000	C77B—C78B	1.3900
C26B—C27B	1.53 (2)	C77B—H77B	0.9500
C26B—H26C	0.9900	C78B—C79B	1.3900
C26B—H26D	0.9900	C78B—H78B	0.9500
C27B—C28B	1.60 (2)	C79B—H79B	0.9500
C27B—H27C	0.9900	C80B—H80D	0.9800
C27B—H27D	0.9900	C80B—H80E	0.9800
C28B—C29B	1.48 (2)	C80B—H80F	0.9800
C28B—H28C	0.9900	C81—C86	1.375 (8)
C28B—H28D	0.9900	C81—C82	1.389 (12)
C29B—C30B	1.53 (2)	C81—C87	1.509 (9)
C29B—H29C	0.9900	C82—C83	1.405 (13)
C29B—H29D	0.9900	C82—H82	0.9500
C30B—H30C	0.9900	C83—C84	1.377 (11)
C30B—H30D	0.9900	C83—H83	0.9500
C31—C36	1.5295 (16)	C84—C85	1.387 (9)
C31—C32	1.5363 (17)	C84—H84	0.9500
C31—H31	1.0000	C85—C86	1.364 (7)
C32—C33	1.5357 (18)	C85—H85	0.9500
C32—H32A	0.9900	C86—H86	0.9500
C32—H32B	0.9900	C87—H87A	0.9800
C33—C34	1.519 (2)	C87—H87B	0.9800
C33—H33A	0.9900	C87—H87C	0.9800
C33—H33B	0.9900		
			
N1—Ti1—N2	110.87 (4)	C30B—C29B—H29C	110.4
N1—Ti1—N3	107.05 (4)	C28B—C29B—H29D	110.4
N2—Ti1—N3	105.94 (4)	C30B—C29B—H29D	110.4
N1—Ti1—O1	109.57 (4)	H29C—C29B—H29D	108.6
N2—Ti1—O1	112.06 (4)	C25B—C30B—C29B	118.2 (12)
N3—Ti1—O1	111.18 (4)	C25B—C30B—H30C	107.8
N1—Ti1—C25B	138.8 (3)	C29B—C30B—H30C	107.8
N2—Ti1—C25B	95.3 (2)	C25B—C30B—H30D	107.8
N3—Ti1—C25B	32.9 (3)	C29B—C30B—H30D	107.8
O1—Ti1—C25B	87.7 (2)	H30C—C30B—H30D	107.1
O1—P1—C37	111.81 (5)	N3—C31—C36	114.31 (10)
O1—P1—C49	110.71 (5)	N3—C31—C32	112.44 (9)
C37—P1—C49	107.56 (5)	C36—C31—C32	110.93 (9)
O1—P1—C43	110.53 (5)	N3—C31—H31	106.2
C37—P1—C43	109.96 (5)	C36—C31—H31	106.2
C49—P1—C43	106.08 (5)	C32—C31—H31	106.2
P1—O1—Ti1	170.99 (5)	C33—C32—C31	110.78 (11)
C7—N1—C1	113.10 (8)	C33—C32—H32A	109.5
C7—N1—Ti1	136.96 (7)	C31—C32—H32A	109.5
C1—N1—Ti1	109.87 (6)	C33—C32—H32B	109.5
C19—N2—C13	121.46 (8)	C31—C32—H32B	109.5
C19—N2—Ti1	129.19 (7)	H32A—C32—H32B	108.1
C13—N2—Ti1	109.09 (6)	C34—C33—C32	111.18 (12)
C25B—N3—C31	134.6 (5)	C34—C33—H33A	109.4
C31—N3—C25A	112.41 (9)	C32—C33—H33A	109.4
C25B—N3—Ti1	102.1 (5)	C34—C33—H33B	109.4
C31—N3—Ti1	123.07 (7)	C32—C33—H33B	109.4
C25A—N3—Ti1	124.52 (8)	H33A—C33—H33B	108.0
N1—C1—C2	113.67 (9)	C35—C34—C33	110.63 (12)
N1—C1—C6	111.52 (9)	C35—C34—H34A	109.5
C2—C1—C6	110.67 (9)	C33—C34—H34A	109.5
N1—C1—H1	106.9	C35—C34—H34B	109.5
C2—C1—H1	106.9	C33—C34—H34B	109.5
C6—C1—H1	106.9	H34A—C34—H34B	108.1
C3—C2—C1	111.65 (11)	C34—C35—C36	111.62 (12)
C3—C2—H2A	109.3	C34—C35—H35A	109.3
C1—C2—H2A	109.3	C36—C35—H35A	109.3
C3—C2—H2B	109.3	C34—C35—H35B	109.3
C1—C2—H2B	109.3	C36—C35—H35B	109.3
H2A—C2—H2B	108.0	H35A—C35—H35B	108.0
C4—C3—C2	111.00 (12)	C31—C36—C35	110.55 (11)
C4—C3—H3A	109.4	C31—C36—H36A	109.5
C2—C3—H3A	109.4	C35—C36—H36A	109.5
C4—C3—H3B	109.4	C31—C36—H36B	109.5
C2—C3—H3B	109.4	C35—C36—H36B	109.5
H3A—C3—H3B	108.0	H36A—C36—H36B	108.1
C3—C4—C5	110.29 (12)	C38—C37—C42	120.06 (10)
C3—C4—H4A	109.6	C38—C37—P1	118.21 (9)
C5—C4—H4A	109.6	C42—C37—P1	121.55 (9)
C3—C4—H4B	109.6	C39—C38—C37	120.02 (12)
C5—C4—H4B	109.6	C39—C38—H38	120.0
H4A—C4—H4B	108.1	C37—C38—H38	120.0
C4—C5—C6	111.58 (11)	C38—C39—C40	119.91 (13)
C4—C5—H5A	109.3	C38—C39—H39	120.0
C6—C5—H5A	109.3	C40—C39—H39	120.0
C4—C5—H5B	109.3	C41—C40—C39	120.19 (12)
C6—C5—H5B	109.3	C41—C40—H40	119.9
H5A—C5—H5B	108.0	C39—C40—H40	119.9
C5—C6—C1	112.98 (10)	C40—C41—C42	120.62 (12)
C5—C6—H6A	109.0	C40—C41—H41	119.7
C1—C6—H6A	109.0	C42—C41—H41	119.7
C5—C6—H6B	109.0	C41—C42—C37	119.19 (12)
C1—C6—H6B	109.0	C41—C42—H42	120.4
H6A—C6—H6B	107.8	C37—C42—H42	120.4
N1—C7—C12	111.05 (9)	C44—C43—C48	119.93 (10)
N1—C7—C8	112.52 (9)	C44—C43—P1	120.26 (8)
C12—C7—C8	110.29 (9)	C48—C43—P1	119.79 (8)
N1—C7—H7	107.6	C45—C44—C43	119.82 (11)
C12—C7—H7	107.6	C45—C44—H44	120.1
C8—C7—H7	107.6	C43—C44—H44	120.1
C7—C8—C9	111.21 (10)	C46—C45—C44	119.83 (11)
C7—C8—H8A	109.4	C46—C45—H45	120.1
C9—C8—H8A	109.4	C44—C45—H45	120.1
C7—C8—H8B	109.4	C47—C46—C45	120.68 (11)
C9—C8—H8B	109.4	C47—C46—H46	119.7
H8A—C8—H8B	108.0	C45—C46—H46	119.7
C10—C9—C8	111.44 (11)	C46—C47—C48	119.85 (12)
C10—C9—H9A	109.3	C46—C47—H47	120.1
C8—C9—H9A	109.3	C48—C47—H47	120.1
C10—C9—H9B	109.3	C47—C48—C43	119.88 (11)
C8—C9—H9B	109.3	C47—C48—H48	120.1
H9A—C9—H9B	108.0	C43—C48—H48	120.1
C9—C10—C11	110.77 (11)	C54—C49—C50	119.81 (10)
C9—C10—H10A	109.5	C54—C49—P1	122.14 (8)
C11—C10—H10A	109.5	C50—C49—P1	117.93 (8)
C9—C10—H10B	109.5	C51—C50—C49	120.17 (10)
C11—C10—H10B	109.5	C51—C50—H50	119.9
H10A—C10—H10B	108.1	C49—C50—H50	119.9
C12—C11—C10	111.16 (11)	C50—C51—C52	119.73 (11)
C12—C11—H11A	109.4	C50—C51—H51	120.1
C10—C11—H11A	109.4	C52—C51—H51	120.1
C12—C11—H11B	109.4	C53—C52—C51	120.38 (11)
C10—C11—H11B	109.4	C53—C52—H52	119.8
H11A—C11—H11B	108.0	C51—C52—H52	119.8
C7—C12—C11	112.24 (10)	C52—C53—C54	120.30 (11)
C7—C12—H12A	109.2	C52—C53—H53	119.8
C11—C12—H12A	109.2	C54—C53—H53	119.8
C7—C12—H12B	109.2	C53—C54—C49	119.56 (11)
C11—C12—H12B	109.2	C53—C54—H54	120.2
H12A—C12—H12B	107.9	C49—C54—H54	120.2
N2—C13—C14	118.24 (8)	C56—C55—C60	112.93 (13)
N2—C13—C18	113.75 (8)	C56—C55—B1	127.02 (12)
C14—C13—C18	108.11 (8)	C60—C55—B1	119.62 (13)
N2—C13—H13	105.2	F1—C56—C55	120.93 (13)
C14—C13—H13	105.2	F1—C56—C57	115.29 (14)
C18—C13—H13	105.2	C55—C56—C57	123.78 (15)
C15—C14—C13	109.09 (9)	F2—C57—C58	120.30 (15)
C15—C14—H14A	109.9	F2—C57—C56	119.89 (16)
C13—C14—H14A	109.9	C58—C57—C56	119.80 (16)
C15—C14—H14B	109.9	F3—C58—C59	120.72 (17)
C13—C14—H14B	109.9	F3—C58—C57	120.26 (18)
H14A—C14—H14B	108.3	C59—C58—C57	119.02 (14)
C16—C15—C14	111.56 (10)	F4—C59—C58	119.65 (15)
C16—C15—H15A	109.3	F4—C59—C60	121.14 (17)
C14—C15—H15A	109.3	C58—C59—C60	119.21 (15)
C16—C15—H15B	109.3	F5—C60—C59	115.31 (14)
C14—C15—H15B	109.3	F5—C60—C55	119.60 (13)
H15A—C15—H15B	108.0	C59—C60—C55	125.09 (15)
C15—C16—C17	111.14 (9)	C66—C61—C62	113.23 (11)
C15—C16—H16A	109.4	C66—C61—B1	120.19 (11)
C17—C16—H16A	109.4	C62—C61—B1	126.56 (12)
C15—C16—H16B	109.4	F6—C62—C63	115.29 (12)
C17—C16—H16B	109.4	F6—C62—C61	120.66 (12)
H16A—C16—H16B	108.0	C63—C62—C61	124.04 (13)
C16—C17—C18	111.22 (9)	F7—C63—C64	119.77 (13)
C16—C17—H17A	109.4	F7—C63—C62	120.45 (14)
C18—C17—H17A	109.4	C64—C63—C62	119.78 (12)
C16—C17—H17B	109.4	F8—C64—C63	121.14 (13)
C18—C17—H17B	109.4	F8—C64—C65	120.19 (14)
H17A—C17—H17B	108.0	C63—C64—C65	118.68 (12)
C17—C18—C13	111.56 (9)	F9—C65—C64	119.33 (12)
C17—C18—H18A	109.3	F9—C65—C66	120.96 (12)
C13—C18—H18A	109.3	C64—C65—C66	119.71 (13)
C17—C18—H18B	109.3	F10—C66—C65	115.29 (12)
C13—C18—H18B	109.3	F10—C66—C61	120.15 (11)
H18A—C18—H18B	108.0	C65—C66—C61	124.55 (12)
N2—C19—C24	117.00 (8)	C68—C67—C72	113.09 (11)
N2—C19—C20	113.57 (8)	C68—C67—B1	124.27 (11)
C24—C19—C20	108.71 (9)	C72—C67—B1	122.64 (11)
N2—C19—H19	105.5	F11—C68—C69	115.05 (12)
C24—C19—H19	105.5	F11—C68—C67	120.43 (11)
C20—C19—H19	105.5	C69—C68—C67	124.51 (12)
C21—C20—C19	110.56 (9)	F12—C69—C70	119.48 (12)
C21—C20—H20A	109.5	F12—C69—C68	121.00 (13)
C19—C20—H20A	109.5	C70—C69—C68	119.51 (13)
C21—C20—H20B	109.5	F13—C70—C71	120.72 (13)
C19—C20—H20B	109.5	F13—C70—C69	120.45 (13)
H20A—C20—H20B	108.1	C71—C70—C69	118.81 (12)
C22—C21—C20	111.17 (10)	F14—C71—C70	119.92 (12)
C22—C21—H21A	109.4	F14—C71—C72	120.29 (14)
C20—C21—H21A	109.4	C70—C71—C72	119.79 (13)
C22—C21—H21B	109.4	F15—C72—C71	114.88 (12)
C20—C21—H21B	109.4	F15—C72—C67	120.83 (12)
H21A—C21—H21B	108.0	C71—C72—C67	124.26 (13)
C21—C22—C23	110.81 (10)	B1—C73—H73A	109.5
C21—C22—H22A	109.5	B1—C73—H73B	109.5
C23—C22—H22A	109.5	H73A—C73—H73B	109.5
C21—C22—H22B	109.5	B1—C73—H73C	109.5
C23—C22—H22B	109.5	H73A—C73—H73C	109.5
H22A—C22—H22B	108.1	H73B—C73—H73C	109.5
C22—C23—C24	111.85 (10)	C73—B1—C55	105.79 (11)
C22—C23—H23A	109.2	C73—B1—C61	108.25 (10)
C24—C23—H23A	109.2	C55—B1—C61	111.81 (10)
C22—C23—H23B	109.2	C73—B1—C67	110.26 (10)
C24—C23—H23B	109.2	C55—B1—C67	111.47 (10)
H23A—C23—H23B	107.9	C61—B1—C67	109.17 (10)
C23—C24—C19	110.05 (9)	C75A—C74A—C79A	116.4 (2)
C23—C24—H24A	109.7	C75A—C74A—C80A	124.4 (3)
C19—C24—H24A	109.7	C79A—C74A—C80A	119.2 (3)
C23—C24—H24B	109.7	C76A—C75A—C74A	122.5 (3)
C19—C24—H24B	109.7	C76A—C75A—H75A	118.8
H24A—C24—H24B	108.2	C74A—C75A—H75A	118.8
N3—C25A—C26A	112.57 (11)	C77A—C76A—C75A	121.5 (3)
N3—C25A—C30A	112.41 (12)	C77A—C76A—H76A	119.3
C26A—C25A—C30A	109.72 (14)	C75A—C76A—H76A	119.3
N3—C25A—H25A	107.3	C78A—C77A—C76A	120.6 (3)
C26A—C25A—H25A	107.3	C78A—C77A—H77A	119.7
C30A—C25A—H25A	107.3	C76A—C77A—H77A	119.7
C25A—C26A—C27A	111.14 (13)	C77A—C78A—C79A	121.3 (3)
C25A—C26A—H26A	109.4	C77A—C78A—H78A	119.4
C27A—C26A—H26A	109.4	C79A—C78A—H78A	119.4
C25A—C26A—H26B	109.4	C78A—C79A—C74A	117.8 (2)
C27A—C26A—H26B	109.4	C78A—C79A—H79A	121.1
H26A—C26A—H26B	108.0	C74A—C79A—H79A	121.1
C28A—C27A—C26A	112.36 (19)	C74A—C80A—H80A	109.5
C28A—C27A—H27A	109.1	C74A—C80A—H80B	109.5
C26A—C27A—H27A	109.1	H80A—C80A—H80B	109.5
C28A—C27A—H27B	109.1	C74A—C80A—H80C	109.5
C26A—C27A—H27B	109.1	H80A—C80A—H80C	109.5
H27A—C27A—H27B	107.9	H80B—C80A—H80C	109.5
C27A—C28A—C29A	110.86 (15)	C80B—C74B—C75B	116.6 (9)
C27A—C28A—H28A	109.5	C80B—C74B—C79B	123.4 (9)
C29A—C28A—H28A	109.5	C75B—C74B—C79B	120.0
C27A—C28A—H28B	109.5	C74B—C75B—C76B	120.0
C29A—C28A—H28B	109.5	C74B—C75B—H75B	120.0
H28A—C28A—H28B	108.1	C76B—C75B—H75B	120.0
C28A—C29A—C30A	111.36 (16)	C77B—C76B—C75B	120.0
C28A—C29A—H29A	109.4	C77B—C76B—H76B	120.0
C30A—C29A—H29A	109.4	C75B—C76B—H76B	120.0
C28A—C29A—H29B	109.4	C76B—C77B—C78B	120.0
C30A—C29A—H29B	109.4	C76B—C77B—H77B	120.0
H29A—C29A—H29B	108.0	C78B—C77B—H77B	120.0
C29A—C30A—C25A	110.52 (16)	C79B—C78B—C77B	120.0
C29A—C30A—H30A	109.5	C79B—C78B—H78B	120.0
C25A—C30A—H30A	109.5	C77B—C78B—H78B	120.0
C29A—C30A—H30B	109.5	C78B—C79B—C74B	120.0
C25A—C30A—H30B	109.5	C78B—C79B—H79B	120.0
H30A—C30A—H30B	108.1	C74B—C79B—H79B	120.0
C30B—C25B—N3	114.7 (10)	C74B—C80B—H80D	109.5
C30B—C25B—C26B	108.0 (12)	C74B—C80B—H80E	109.5
N3—C25B—C26B	113.5 (9)	H80D—C80B—H80E	109.5
C30B—C25B—Ti1	116.8 (9)	C74B—C80B—H80F	109.5
N3—C25B—Ti1	44.9 (3)	H80D—C80B—H80F	109.5
C26B—C25B—Ti1	135.1 (7)	H80E—C80B—H80F	109.5
C30B—C25B—H25B	106.7	C86—C81—C82	117.0 (7)
N3—C25B—H25B	106.7	C86—C81—C87	120.2 (6)
C26B—C25B—H25B	106.7	C82—C81—C87	122.8 (7)
Ti1—C25B—H25B	63.1	C81—C82—C83	120.4 (9)
C25B—C26B—C27B	114.3 (11)	C81—C82—H82	119.8
C25B—C26B—H26C	108.7	C83—C82—H82	119.8
C27B—C26B—H26C	108.7	C84—C83—C82	120.2 (7)
C25B—C26B—H26D	108.7	C84—C83—H83	119.9
C27B—C26B—H26D	108.7	C82—C83—H83	119.9
H26C—C26B—H26D	107.6	C83—C84—C85	119.6 (6)
C26B—C27B—C28B	104.7 (14)	C83—C84—H84	120.2
C26B—C27B—H27C	110.8	C85—C84—H84	120.2
C28B—C27B—H27C	110.8	C86—C85—C84	118.9 (6)
C26B—C27B—H27D	110.8	C86—C85—H85	120.6
C28B—C27B—H27D	110.8	C84—C85—H85	120.6
H27C—C27B—H27D	108.9	C85—C86—C81	123.9 (7)
C29B—C28B—C27B	115.4 (14)	C85—C86—H86	118.0
C29B—C28B—H28C	108.4	C81—C86—H86	118.0
C27B—C28B—H28C	108.4	C81—C87—H87A	109.5
C29B—C28B—H28D	108.4	C81—C87—H87B	109.5
C27B—C28B—H28D	108.4	H87A—C87—H87B	109.5
H28C—C28B—H28D	107.5	C81—C87—H87C	109.5
C28B—C29B—C30B	106.6 (13)	H87A—C87—H87C	109.5
C28B—C29B—H29C	110.4	H87B—C87—H87C	109.5
			
C7—N1—C1—C2	58.41 (12)	C54—C49—C50—C51	2.08 (18)
Ti1—N1—C1—C2	−124.07 (8)	P1—C49—C50—C51	177.97 (9)
C7—N1—C1—C6	−67.55 (11)	C49—C50—C51—C52	−0.50 (19)
Ti1—N1—C1—C6	109.97 (8)	C50—C51—C52—C53	−1.16 (19)
N1—C1—C2—C3	−179.98 (10)	C51—C52—C53—C54	1.3 (2)
C6—C1—C2—C3	−53.58 (14)	C52—C53—C54—C49	0.3 (2)
C1—C2—C3—C4	58.10 (15)	C50—C49—C54—C53	−1.98 (18)
C2—C3—C4—C5	−58.42 (17)	P1—C49—C54—C53	−177.70 (10)
C3—C4—C5—C6	55.58 (17)	C60—C55—C56—F1	−176.31 (13)
C4—C5—C6—C1	−52.73 (15)	B1—C55—C56—F1	11.3 (2)
N1—C1—C6—C5	178.78 (9)	C60—C55—C56—C57	4.0 (2)
C2—C1—C6—C5	51.19 (13)	B1—C55—C56—C57	−168.41 (14)
C1—N1—C7—C12	130.48 (10)	F1—C56—C57—F2	−2.1 (2)
Ti1—N1—C7—C12	−46.11 (14)	C55—C56—C57—F2	177.57 (14)
C1—N1—C7—C8	−105.34 (10)	F1—C56—C57—C58	179.28 (14)
Ti1—N1—C7—C8	78.07 (13)	C55—C56—C57—C58	−1.0 (2)
N1—C7—C8—C9	179.58 (10)	F2—C57—C58—F3	−0.7 (2)
C12—C7—C8—C9	−55.82 (13)	C56—C57—C58—F3	177.93 (14)
C7—C8—C9—C10	56.39 (14)	F2—C57—C58—C59	179.01 (15)
C8—C9—C10—C11	−55.34 (15)	C56—C57—C58—C59	−2.4 (2)
C9—C10—C11—C12	54.50 (15)	F3—C58—C59—F4	2.8 (2)
N1—C7—C12—C11	−178.84 (10)	C57—C58—C59—F4	−176.89 (14)
C8—C7—C12—C11	55.73 (13)	F3—C58—C59—C60	−177.93 (14)
C10—C11—C12—C7	−55.42 (14)	C57—C58—C59—C60	2.4 (2)
C19—N2—C13—C14	51.99 (13)	F4—C59—C60—F5	1.2 (2)
Ti1—N2—C13—C14	−133.41 (8)	C58—C59—C60—F5	−178.02 (13)
C19—N2—C13—C18	−76.47 (11)	F4—C59—C60—C55	−179.72 (14)
Ti1—N2—C13—C18	98.14 (8)	C58—C59—C60—C55	1.0 (2)
N2—C13—C14—C15	168.15 (9)	C56—C55—C60—F5	174.93 (13)
C18—C13—C14—C15	−60.80 (11)	B1—C55—C60—F5	−12.0 (2)
C13—C14—C15—C16	59.84 (12)	C56—C55—C60—C59	−4.1 (2)
C14—C15—C16—C17	−55.31 (13)	B1—C55—C60—C59	168.98 (13)
C15—C16—C17—C18	52.62 (13)	C66—C61—C62—F6	−177.12 (12)
C16—C17—C18—C13	−55.87 (13)	B1—C61—C62—F6	4.5 (2)
N2—C13—C18—C17	−166.78 (9)	C66—C61—C62—C63	1.5 (2)
C14—C13—C18—C17	59.77 (11)	B1—C61—C62—C63	−176.94 (13)
C13—N2—C19—C24	45.86 (13)	F6—C62—C63—F7	−1.5 (2)
Ti1—N2—C19—C24	−127.55 (9)	C61—C62—C63—F7	179.83 (13)
C13—N2—C19—C20	−82.09 (11)	F6—C62—C63—C64	178.06 (13)
Ti1—N2—C19—C20	104.49 (10)	C61—C62—C63—C64	−0.6 (2)
N2—C19—C20—C21	−168.06 (9)	F7—C63—C64—F8	0.0 (2)
C24—C19—C20—C21	59.82 (11)	C62—C63—C64—F8	−179.56 (13)
C19—C20—C21—C22	−57.77 (13)	F7—C63—C64—C65	179.52 (13)
C20—C21—C22—C23	54.34 (14)	C62—C63—C64—C65	−0.1 (2)
C21—C22—C23—C24	−54.56 (14)	F8—C64—C65—F9	−0.36 (19)
C22—C23—C24—C19	57.59 (14)	C63—C64—C65—F9	−179.87 (12)
N2—C19—C24—C23	170.40 (9)	F8—C64—C65—C66	179.21 (12)
C20—C19—C24—C23	−59.33 (12)	C63—C64—C65—C66	−0.30 (19)
C31—N3—C25A—C26A	132.12 (13)	F9—C65—C66—F10	2.28 (17)
Ti1—N3—C25A—C26A	−47.27 (15)	C64—C65—C66—F10	−177.28 (11)
C31—N3—C25A—C30A	−103.37 (15)	F9—C65—C66—C61	−179.08 (11)
Ti1—N3—C25A—C30A	77.24 (15)	C64—C65—C66—C61	1.36 (19)
N3—C25A—C26A—C27A	−178.33 (17)	C62—C61—C66—F10	176.72 (11)
C30A—C25A—C26A—C27A	55.7 (2)	B1—C61—C66—F10	−4.76 (17)
C25A—C26A—C27A—C28A	−55.3 (2)	C62—C61—C66—C65	−1.86 (18)
C26A—C27A—C28A—C29A	54.7 (2)	B1—C61—C66—C65	176.66 (11)
C27A—C28A—C29A—C30A	−55.9 (2)	C72—C67—C68—F11	−176.85 (12)
C28A—C29A—C30A—C25A	57.6 (2)	B1—C67—C68—F11	3.82 (19)
N3—C25A—C30A—C29A	176.84 (15)	C72—C67—C68—C69	1.88 (19)
C26A—C25A—C30A—C29A	−57.1 (2)	B1—C67—C68—C69	−177.45 (12)
C31—N3—C25B—C30B	−81.1 (12)	F11—C68—C69—F12	−2.89 (19)
Ti1—N3—C25B—C30B	104.0 (11)	C67—C68—C69—F12	178.32 (12)
C31—N3—C25B—C26B	43.7 (12)	F11—C68—C69—C70	178.23 (12)
Ti1—N3—C25B—C26B	−131.2 (9)	C67—C68—C69—C70	−0.6 (2)
C31—N3—C25B—Ti1	174.9 (5)	F12—C69—C70—F13	−0.92 (19)
C30B—C25B—C26B—C27B	−58.3 (16)	C68—C69—C70—F13	177.98 (12)
N3—C25B—C26B—C27B	173.4 (11)	F12—C69—C70—C71	−179.49 (12)
Ti1—C25B—C26B—C27B	124.5 (12)	C68—C69—C70—C71	−0.59 (19)
C25B—C26B—C27B—C28B	55.6 (16)	F13—C70—C71—F14	0.4 (2)
C26B—C27B—C28B—C29B	−54.7 (19)	C69—C70—C71—F14	178.99 (13)
C27B—C28B—C29B—C30B	52.4 (18)	F13—C70—C71—C72	−178.30 (12)
N3—C25B—C30B—C29B	−175.2 (11)	C69—C70—C71—C72	0.3 (2)
C26B—C25B—C30B—C29B	57.2 (17)	F14—C71—C72—F15	4.2 (2)
Ti1—C25B—C30B—C29B	−125.0 (12)	C70—C71—C72—F15	−177.06 (13)
C28B—C29B—C30B—C25B	−55.2 (19)	F14—C71—C72—C67	−177.46 (13)
C25B—N3—C31—C36	62.0 (6)	C70—C71—C72—C67	1.3 (2)
C25A—N3—C31—C36	56.63 (13)	C68—C67—C72—F15	176.00 (12)
Ti1—N3—C31—C36	−123.97 (9)	B1—C67—C72—F15	−4.7 (2)
C25B—N3—C31—C32	−65.6 (6)	C68—C67—C72—C71	−2.2 (2)
C25A—N3—C31—C32	−71.00 (12)	B1—C67—C72—C71	177.11 (13)
Ti1—N3—C31—C32	108.39 (9)	C56—C55—B1—C73	119.27 (15)
N3—C31—C32—C33	−175.13 (10)	C60—C55—B1—C73	−52.70 (15)
C36—C31—C32—C33	55.46 (14)	C56—C55—B1—C61	−123.10 (14)
C31—C32—C33—C34	−56.05 (16)	C60—C55—B1—C61	64.93 (16)
C32—C33—C34—C35	56.64 (18)	C56—C55—B1—C67	−0.60 (18)
C33—C34—C35—C36	−57.00 (17)	C60—C55—B1—C67	−172.57 (11)
N3—C31—C36—C35	176.15 (10)	C66—C61—B1—C73	−47.07 (15)
C32—C31—C36—C35	−55.44 (14)	C62—C61—B1—C73	131.24 (14)
C34—C35—C36—C31	56.51 (15)	C66—C61—B1—C55	−163.21 (11)
O1—P1—C37—C38	50.49 (10)	C62—C61—B1—C55	15.09 (18)
C49—P1—C37—C38	−71.25 (10)	C66—C61—B1—C67	72.97 (14)
C43—P1—C37—C38	173.67 (9)	C62—C61—B1—C67	−108.72 (14)
O1—P1—C37—C42	−134.34 (9)	C68—C67—B1—C73	148.88 (12)
C49—P1—C37—C42	103.92 (10)	C72—C67—B1—C73	−30.38 (17)
C43—P1—C37—C42	−11.16 (11)	C68—C67—B1—C55	−93.93 (14)
C42—C37—C38—C39	−0.81 (18)	C72—C67—B1—C55	86.80 (15)
P1—C37—C38—C39	174.43 (10)	C68—C67—B1—C61	30.08 (16)
C37—C38—C39—C40	1.0 (2)	C72—C67—B1—C61	−149.18 (12)
C38—C39—C40—C41	−0.4 (2)	C79A—C74A—C75A—C76A	0.1 (3)
C39—C40—C41—C42	−0.4 (2)	C80A—C74A—C75A—C76A	−177.6 (3)
C40—C41—C42—C37	0.64 (19)	C74A—C75A—C76A—C77A	0.8 (4)
C38—C37—C42—C41	−0.02 (17)	C75A—C76A—C77A—C78A	−1.5 (4)
P1—C37—C42—C41	−175.10 (9)	C76A—C77A—C78A—C79A	1.2 (4)
O1—P1—C43—C44	34.36 (10)	C77A—C78A—C79A—C74A	−0.2 (3)
C37—P1—C43—C44	−89.57 (9)	C75A—C74A—C79A—C78A	−0.4 (3)
C49—P1—C43—C44	154.41 (9)	C80A—C74A—C79A—C78A	177.4 (2)
O1—P1—C43—C48	−144.50 (9)	C80B—C74B—C75B—C76B	179.4 (11)
C37—P1—C43—C48	91.57 (10)	C79B—C74B—C75B—C76B	0.0
C49—P1—C43—C48	−24.44 (10)	C74B—C75B—C76B—C77B	0.0
C48—C43—C44—C45	0.92 (16)	C75B—C76B—C77B—C78B	0.0
P1—C43—C44—C45	−177.93 (9)	C76B—C77B—C78B—C79B	0.0
C43—C44—C45—C46	−0.72 (17)	C77B—C78B—C79B—C74B	0.0
C44—C45—C46—C47	0.05 (18)	C80B—C74B—C79B—C78B	−179.4 (12)
C45—C46—C47—C48	0.42 (19)	C75B—C74B—C79B—C78B	0.0
C46—C47—C48—C43	−0.21 (19)	C86—C81—C82—C83	1.3 (14)
C44—C43—C48—C47	−0.46 (17)	C87—C81—C82—C83	−179.7 (8)
P1—C43—C48—C47	178.40 (9)	C81—C82—C83—C84	−1.3 (13)
O1—P1—C49—C54	−126.81 (9)	C82—C83—C84—C85	−0.3 (11)
C37—P1—C49—C54	−4.38 (11)	C83—C84—C85—C86	1.8 (10)
C43—P1—C49—C54	113.25 (10)	C84—C85—C86—C81	−1.7 (10)
O1—P1—C49—C50	57.40 (10)	C82—C81—C86—C85	0.2 (13)
C37—P1—C49—C50	179.83 (9)	C87—C81—C86—C85	−178.8 (7)
C43—P1—C49—C50	−62.54 (10)		

### Tris(dicyclohexylamido)(triphenylphosphine oxide)titanium methyltris(pentafluorophenyl)borate toluene sesquisolvate (1) Hydrogen-bond geometry (Å, º)

**Table:**

*D*—H···*A*	*D*—H	H···*A*	*D*···*A*	*D*—H···*A*
C28*B*—H28*C*···F11^i^	0.99	2.27	2.962 (19)	126
C39—H39···F4^ii^	0.95	2.50	3.2194 (16)	133
C76*A*—H76*A*···F5	0.95	2.45	3.376 (3)	165
C84—H84···F1^iii^	0.95	2.45	3.331 (9)	154
C26*B*—H26*D*···F6^i^	0.99	2.55	3.446 (13)	150
C35—H35*A*···F3^i^	0.99	2.55	3.4738 (18)	155

Symmetry codes: (i) *x*−1, *y*+1, *z*; (ii) −*x*+1, −*y*+1, −*z*; (iii) *x*−1, *y*, *z*.

### Tris(dicyclohexylamido)(4-fluorobenzonitrile)titanium methyltris(pentafluorophenyl)borate toluene sesquisolvate (2) Crystal data

**Table:**

[Ti(C_12_H_22_N)_3_(C_7_H_4_FN)](C_19_H_3_F_15_B)·1.5C_7_H_8_	*Z* = 2
*M**_r_* = 1375.15	*F*(000) = 1438
Triclinic, *P*1	*D*_x_ = 1.372 Mg m^−^^3^
*a* = 12.1151 (5) Å	Mo *K*α radiation, λ = 0.71073 Å
*b* = 13.2796 (6) Å	Cell parameters from 9902 reflections
*c* = 21.1662 (10) Å	θ = 2.3–33.4°
α = 77.9160 (19)°	µ = 0.22 mm^−^^1^
β = 88.8857 (18)°	*T* = 100 K
γ = 87.9827 (18)°	Plate, yellow
*V* = 3327.5 (3) Å^3^	0.32 × 0.30 × 0.08 mm

### Tris(dicyclohexylamido)(4-fluorobenzonitrile)titanium methyltris(pentafluorophenyl)borate toluene sesquisolvate (2) Data collection

**Table:**

Bruker APEXII CCD diffractometer	20132 reflections with *I* > 2σ(*I*)
Radiation source: sealed tube	*R*_int_ = 0.039
φ and ω scans	θ_max_ = 33.7°, θ_min_ = 1.6°
Absorption correction: multi-scan (*SADABS*; Krause *et al.*, 2015)	*h* = −18→18
*T*_min_ = 0.963, *T*_max_ = 1.000	*k* = −20→20
146764 measured reflections	*l* = −33→33
26559 independent reflections	

### Tris(dicyclohexylamido)(4-fluorobenzonitrile)titanium methyltris(pentafluorophenyl)borate toluene sesquisolvate (2) Refinement

**Table:**

Refinement on *F*^2^	Primary atom site location: structure-invariant direct methods
Least-squares matrix: full	Secondary atom site location: difference Fourier map
*R*[*F*^2^ > 2σ(*F*^2^)] = 0.044	Hydrogen site location: difference Fourier map
*wR*(*F*^2^) = 0.118	H-atom parameters constrained
*S* = 1.02	*w* = 1/[σ^2^(*F*_o_^2^) + (0.050*P*)^2^ + 1.3*P*] where *P* = (*F*_o_^2^ + 2*F*_c_^2^)/3
26559 reflections	(Δ/σ)_max_ = 0.002
979 parameters	Δρ_max_ = 0.74 e Å^−^^3^
0 restraints	Δρ_min_ = −0.96 e Å^−^^3^

### Tris(dicyclohexylamido)(4-fluorobenzonitrile)titanium methyltris(pentafluorophenyl)borate toluene sesquisolvate (2) Special details

**Table:**

Geometry. All esds (except the esd in the dihedral angle between two l.s. planes) are estimated using the full covariance matrix. The cell esds are taken into account individually in the estimation of esds in distances, angles and torsion angles; correlations between esds in cell parameters are only used when they are defined by crystal symmetry. An approximate (isotropic) treatment of cell esds is used for estimating esds involving l.s. planes.

### Tris(dicyclohexylamido)(4-fluorobenzonitrile)titanium methyltris(pentafluorophenyl)borate toluene sesquisolvate (2) Fractional atomic coordinates and isotropic or equivalent isotropic displacement parameters (Å^2^)

**Table:**

	*x*	*y*	*z*	*U*_iso_*/*U*_eq_	Occ. (<1)
Ti1	0.58769 (2)	0.47178 (2)	0.24352 (2)	0.01472 (4)	
F1	0.63527 (7)	−0.13875 (6)	0.50255 (4)	0.02993 (16)	
N1	0.54158 (7)	0.56582 (7)	0.29536 (5)	0.01976 (17)	
N2	0.72127 (7)	0.51407 (7)	0.20037 (4)	0.01562 (15)	
N3	0.47906 (7)	0.45031 (7)	0.18509 (5)	0.01827 (16)	
N4	0.60937 (7)	0.32435 (7)	0.30911 (4)	0.01842 (16)	
C1	0.45871 (8)	0.51014 (8)	0.34063 (5)	0.01768 (18)	
H1	0.440369	0.448600	0.323002	0.021*	
C2	0.34831 (10)	0.56871 (10)	0.34577 (6)	0.0240 (2)	
H2A	0.360323	0.628394	0.365796	0.029*	
H2B	0.318311	0.595007	0.302026	0.029*	
C3	0.26549 (10)	0.49736 (11)	0.38668 (6)	0.0284 (2)	
H3A	0.248517	0.441402	0.364320	0.034*	
H3B	0.195938	0.536874	0.391053	0.034*	
C4	0.30984 (12)	0.45011 (12)	0.45407 (6)	0.0306 (3)	
H4A	0.317935	0.505134	0.478641	0.037*	
H4B	0.256491	0.400568	0.477696	0.037*	
C5	0.42158 (11)	0.39479 (10)	0.44942 (6)	0.0260 (2)	
H5A	0.451247	0.369402	0.493331	0.031*	
H5B	0.411874	0.334587	0.429509	0.031*	
C6	0.50330 (10)	0.46718 (9)	0.40898 (6)	0.0216 (2)	
H6A	0.574495	0.429518	0.405824	0.026*	
H6B	0.516805	0.524952	0.430476	0.026*	
C7A	0.56873 (11)	0.66624 (10)	0.31190 (7)	0.0154 (3)	0.764 (2)
H7A	0.503410	0.689838	0.335210	0.018*	0.764 (2)
C8A	0.66928 (12)	0.66100 (11)	0.35534 (7)	0.0179 (3)	0.764 (2)
H8AA	0.658329	0.608521	0.395506	0.022*	0.764 (2)
H8AB	0.736066	0.640462	0.332817	0.022*	0.764 (2)
C9A	0.68535 (13)	0.76606 (12)	0.37206 (7)	0.0224 (3)	0.764 (2)
H9AA	0.620205	0.784542	0.396740	0.027*	0.764 (2)
H9AB	0.751039	0.762216	0.399670	0.027*	0.764 (2)
C10A	0.70061 (13)	0.84922 (13)	0.31096 (8)	0.0236 (3)	0.764 (2)
H10A	0.705577	0.917085	0.322925	0.028*	0.764 (2)
H10B	0.770677	0.835078	0.289082	0.028*	0.764 (2)
C11A	0.60496 (14)	0.85292 (12)	0.26452 (9)	0.0260 (3)	0.764 (2)
H11A	0.620895	0.902621	0.223804	0.031*	0.764 (2)
H11B	0.536733	0.877347	0.283895	0.031*	0.764 (2)
C12A	0.58642 (13)	0.74688 (11)	0.24956 (7)	0.0218 (3)	0.764 (2)
H12A	0.651310	0.725838	0.225669	0.026*	0.764 (2)
H12B	0.520971	0.750912	0.221753	0.026*	0.764 (2)
C7B	0.5886 (4)	0.6714 (3)	0.2773 (2)	0.0164 (8)*	0.236 (2)
H7B	0.654473	0.667445	0.248598	0.020*	0.236 (2)
C8B	0.6282 (4)	0.6972 (4)	0.3397 (2)	0.0211 (9)*	0.236 (2)
H8BA	0.681595	0.643090	0.360956	0.025*	0.236 (2)
H8BB	0.564658	0.699364	0.369620	0.025*	0.236 (2)
C9B	0.6840 (5)	0.8033 (5)	0.3249 (3)	0.0253 (11)*	0.236 (2)
H9BA	0.703632	0.822863	0.365771	0.030*	0.236 (2)
H9BB	0.752787	0.798721	0.299410	0.030*	0.236 (2)
C10B	0.6051 (5)	0.8849 (5)	0.2868 (3)	0.0310 (12)*	0.236 (2)
H10C	0.540627	0.894511	0.314671	0.037*	0.236 (2)
H10D	0.642983	0.951218	0.275477	0.037*	0.236 (2)
C11B	0.5647 (5)	0.8578 (4)	0.2259 (3)	0.0281 (11)*	0.236 (2)
H11C	0.627698	0.854415	0.195799	0.034*	0.236 (2)
H11D	0.511351	0.911742	0.204380	0.034*	0.236 (2)
C12B	0.5091 (4)	0.7538 (4)	0.2419 (2)	0.0223 (10)*	0.236 (2)
H12C	0.442666	0.758751	0.269196	0.027*	0.236 (2)
H12D	0.485360	0.734877	0.201504	0.027*	0.236 (2)
C13	0.80210 (8)	0.48138 (8)	0.25293 (5)	0.01440 (16)	
H13	0.758001	0.473260	0.294018	0.017*	
C14	0.86028 (9)	0.37583 (8)	0.25480 (5)	0.01756 (18)	
H14A	0.805163	0.324734	0.249981	0.021*	
H14B	0.914089	0.381180	0.218540	0.021*	
C15	0.92010 (9)	0.34038 (9)	0.31909 (6)	0.0213 (2)	
H15A	0.865499	0.331713	0.355075	0.026*	
H15B	0.957925	0.272796	0.319978	0.026*	
C16	1.00473 (10)	0.41844 (9)	0.32836 (6)	0.0253 (2)	
H16A	1.065666	0.418585	0.296493	0.030*	
H16B	1.036365	0.397295	0.372096	0.030*	
C17	0.95394 (10)	0.52728 (9)	0.32026 (6)	0.0245 (2)	
H17A	1.013528	0.576274	0.320645	0.029*	
H17B	0.903890	0.530316	0.357397	0.029*	
C18	0.88909 (8)	0.56064 (8)	0.25748 (5)	0.01730 (18)	
H18A	0.940483	0.567176	0.220010	0.021*	
H18B	0.852344	0.628766	0.256333	0.021*	
C19	0.76204 (9)	0.56945 (8)	0.13712 (5)	0.01761 (18)	
H19	0.815620	0.620819	0.145172	0.021*	
C20	0.66669 (9)	0.62922 (8)	0.09867 (5)	0.01969 (19)	
H20A	0.631965	0.677333	0.123702	0.024*	
H20B	0.610184	0.580436	0.092109	0.024*	
C21	0.70539 (12)	0.69013 (10)	0.03292 (6)	0.0263 (2)	
H21A	0.641073	0.725578	0.008716	0.032*	
H21B	0.757016	0.743187	0.039309	0.032*	
C22	0.76286 (11)	0.61869 (11)	−0.00592 (6)	0.0281 (2)	
H22A	0.709507	0.569133	−0.015308	0.034*	
H22B	0.789769	0.659608	−0.047588	0.034*	
C23	0.85958 (11)	0.56022 (13)	0.03128 (6)	0.0324 (3)	
H23A	0.916043	0.609584	0.036983	0.039*	
H23B	0.893684	0.512110	0.006036	0.039*	
C24	0.82299 (10)	0.49925 (10)	0.09787 (6)	0.0246 (2)	
H24A	0.773750	0.443991	0.092139	0.030*	
H24B	0.888637	0.466436	0.121932	0.030*	
C25	0.52184 (9)	0.35424 (9)	0.16646 (5)	0.01896 (19)	
H25	0.597363	0.341290	0.185299	0.023*	
C26	0.53797 (11)	0.35895 (10)	0.09370 (6)	0.0254 (2)	
H26A	0.465113	0.365339	0.072594	0.030*	
H26B	0.580486	0.420281	0.074137	0.030*	
C27	0.59979 (13)	0.26151 (12)	0.08260 (7)	0.0348 (3)	
H27A	0.606521	0.264159	0.035591	0.042*	
H27B	0.675255	0.259074	0.100092	0.042*	
C28	0.54091 (15)	0.16428 (12)	0.11476 (8)	0.0403 (3)	
H28A	0.586739	0.103131	0.109840	0.048*	
H28B	0.469871	0.161735	0.092897	0.048*	
C29	0.51895 (13)	0.16058 (10)	0.18651 (8)	0.0344 (3)	
H29A	0.590038	0.153385	0.209575	0.041*	
H29B	0.474895	0.099740	0.204940	0.041*	
C30	0.45669 (10)	0.25831 (9)	0.19645 (6)	0.0243 (2)	
H30A	0.445384	0.255274	0.243252	0.029*	
H30B	0.383225	0.262887	0.176175	0.029*	
C31	0.37318 (9)	0.49231 (10)	0.15624 (6)	0.0235 (2)	
H31A	0.345103	0.438981	0.134058	0.028*	0.146 (3)
H31	0.368796	0.474054	0.112875	0.028*	0.854 (3)
C32A	0.36726 (13)	0.61028 (13)	0.14610 (11)	0.0417 (5)	0.854 (3)
H32A	0.369964	0.630334	0.188523	0.050*	0.854 (3)
H32B	0.432003	0.639139	0.120149	0.050*	0.854 (3)
C33A	0.26110 (14)	0.65527 (19)	0.11151 (13)	0.0558 (7)	0.854 (3)
H33A	0.257855	0.730902	0.107654	0.067*	0.854 (3)
H33B	0.262027	0.641290	0.067352	0.067*	0.854 (3)
C34A	0.15872 (13)	0.60892 (16)	0.14802 (10)	0.0380 (4)	0.854 (3)
H34A	0.151578	0.631812	0.189561	0.046*	0.854 (3)
H34B	0.092168	0.634090	0.122301	0.046*	0.854 (3)
C35A	0.16544 (11)	0.49289 (15)	0.16092 (9)	0.0327 (4)	0.854 (3)
H35A	0.162528	0.469919	0.119420	0.039*	0.854 (3)
H35B	0.100953	0.465170	0.187563	0.039*	0.854 (3)
C36A	0.27137 (11)	0.44999 (12)	0.19583 (8)	0.0259 (3)	0.854 (3)
H36A	0.273532	0.373862	0.202917	0.031*	0.854 (3)
H36B	0.272317	0.469121	0.238578	0.031*	0.854 (3)
C32B	0.3846 (7)	0.5883 (6)	0.1046 (4)	0.0232 (17)*	0.146 (3)
H32C	0.433666	0.572991	0.069662	0.028*	0.146 (3)
H32D	0.419377	0.641784	0.123210	0.028*	0.146 (3)
C33B	0.2725 (9)	0.6299 (8)	0.0763 (5)	0.032 (2)*	0.146 (3)
H33C	0.282102	0.695461	0.044603	0.039*	0.146 (3)
H33D	0.240592	0.579831	0.053626	0.039*	0.146 (3)
C34B	0.1980 (11)	0.6470 (9)	0.1288 (5)	0.035 (2)*	0.146 (3)
H34C	0.126010	0.675148	0.110407	0.042*	0.146 (3)
H34D	0.229599	0.698653	0.150260	0.042*	0.146 (3)
C35B	0.1794 (8)	0.5469 (8)	0.1792 (5)	0.030 (2)*	0.146 (3)
H35C	0.141737	0.496759	0.159142	0.036*	0.146 (3)
H35D	0.132414	0.561669	0.215183	0.036*	0.146 (3)
C36B	0.2943 (9)	0.5015 (9)	0.2052 (5)	0.034 (2)*	0.146 (3)
H36C	0.323952	0.546161	0.232468	0.040*	0.146 (3)
H36D	0.284092	0.432550	0.232990	0.040*	0.146 (3)
C37	0.61583 (8)	0.24326 (8)	0.34115 (5)	0.01769 (18)	
C38	0.62239 (8)	0.14243 (8)	0.38129 (5)	0.01631 (17)	
C39	0.53396 (9)	0.07706 (9)	0.38354 (6)	0.0204 (2)	
H39	0.471098	0.098276	0.357357	0.025*	
C40	0.53907 (9)	−0.01897 (9)	0.42438 (6)	0.0209 (2)	
H40	0.480094	−0.064962	0.426757	0.025*	
C41	0.63184 (9)	−0.04632 (8)	0.46155 (5)	0.01995 (19)	
C42	0.72100 (10)	0.01632 (9)	0.45953 (6)	0.0260 (2)	
H42	0.783815	−0.005819	0.485585	0.031*	
C43	0.71624 (9)	0.11212 (9)	0.41852 (6)	0.0234 (2)	
H43	0.776364	0.156944	0.415716	0.028*	
F2	−0.05179 (6)	0.76843 (6)	0.35769 (4)	0.02630 (15)	
F3	−0.04263 (7)	0.61787 (6)	0.46154 (4)	0.03156 (17)	
F4	0.14075 (8)	0.58544 (7)	0.53696 (4)	0.0379 (2)	
F5	0.31575 (8)	0.70966 (7)	0.50452 (5)	0.0405 (2)	
F6	0.30875 (6)	0.86273 (6)	0.40161 (4)	0.03312 (18)	
F7	−0.03531 (5)	0.97154 (5)	0.40045 (3)	0.02145 (13)	
F8	−0.03525 (6)	1.11860 (6)	0.46882 (4)	0.02597 (15)	
F9	0.13222 (7)	1.25359 (6)	0.45417 (4)	0.03056 (17)	
F10	0.29578 (7)	1.24196 (6)	0.36392 (4)	0.03015 (16)	
F11	0.29884 (6)	1.09705 (6)	0.29689 (4)	0.02678 (15)	
F12	−0.05031 (6)	1.09714 (6)	0.27387 (4)	0.02802 (15)	
F13	−0.19995 (7)	1.10682 (7)	0.18506 (4)	0.0403 (2)	
F14	−0.21436 (8)	0.95434 (8)	0.11699 (4)	0.0469 (2)	
F15	−0.07651 (9)	0.78476 (7)	0.14632 (4)	0.0453 (2)	
F16	0.07024 (8)	0.76763 (6)	0.24002 (4)	0.03439 (18)	
C44	0.12937 (9)	0.82299 (8)	0.37139 (6)	0.01900 (19)	
C45	0.04288 (9)	0.75696 (9)	0.39110 (6)	0.02016 (19)	
C46	0.04444 (10)	0.67803 (9)	0.44575 (6)	0.0233 (2)	
C47	0.13671 (11)	0.66154 (9)	0.48412 (6)	0.0264 (2)	
C48	0.22456 (11)	0.72453 (10)	0.46742 (6)	0.0273 (2)	
C49	0.21914 (9)	0.80237 (9)	0.41286 (6)	0.0233 (2)	
C50	0.13296 (8)	1.02259 (8)	0.34589 (5)	0.01730 (18)	
C51	0.05085 (8)	1.03495 (8)	0.39121 (5)	0.01742 (18)	
C52	0.04843 (9)	1.11076 (8)	0.42697 (5)	0.01913 (19)	
C53	0.13194 (10)	1.18093 (8)	0.41886 (6)	0.0214 (2)	
C54	0.21402 (9)	1.17386 (8)	0.37400 (6)	0.0211 (2)	
C55	0.21319 (9)	1.09622 (9)	0.33926 (5)	0.01928 (19)	
C56	0.02351 (9)	0.93270 (9)	0.26149 (5)	0.01989 (19)	
C57	−0.05082 (10)	1.01562 (9)	0.24457 (6)	0.0220 (2)	
C58	−0.13061 (10)	1.02409 (11)	0.19734 (6)	0.0280 (2)	
C59	−0.13849 (12)	0.94712 (12)	0.16341 (6)	0.0324 (3)	
C60	−0.06861 (12)	0.86179 (11)	0.17846 (6)	0.0310 (3)	
C61	0.00824 (11)	0.85585 (10)	0.22664 (6)	0.0255 (2)	
C62	0.24065 (11)	0.91266 (11)	0.26398 (7)	0.0307 (3)	
H62A	0.307621	0.915690	0.288662	0.046*	
H62B	0.239339	0.969189	0.225880	0.046*	
H62C	0.240187	0.846660	0.250211	0.046*	
B1	0.13144 (10)	0.92343 (10)	0.30953 (6)	0.0191 (2)	
C63A	1.0671 (2)	0.1870 (2)	0.11163 (15)	0.0357 (6)	0.5
C64A	1.1126 (5)	0.1153 (6)	0.0784 (3)	0.0540 (15)	0.5
H64A	1.180367	0.080166	0.092217	0.065*	0.5
C65A	1.0589 (4)	0.0948 (4)	0.0248 (2)	0.0676 (13)	0.5
H65A	1.090918	0.046012	0.002308	0.081*	0.5
C66A	0.9611 (4)	0.1440 (4)	0.0044 (2)	0.0560 (10)	0.5
H66A	0.925299	0.130030	−0.032235	0.067*	0.5
C67A	0.9149 (7)	0.2148 (7)	0.0380 (3)	0.0579 (16)	0.5
H67A	0.845935	0.248576	0.025325	0.070*	0.5
C68A	0.9692 (5)	0.2354 (4)	0.0893 (2)	0.0476 (11)	0.5
H68A	0.937818	0.285859	0.110801	0.057*	0.5
C69A	1.1258 (5)	0.2084 (5)	0.1680 (3)	0.0561 (15)	0.5
H69A	1.138677	0.143906	0.199579	0.084*	0.5
H69B	1.080809	0.256357	0.187902	0.084*	0.5
H69C	1.196801	0.239092	0.153623	0.084*	0.5
C63B	1.0041 (3)	0.1871 (3)	0.06176 (16)	0.0400 (7)	0.5
C64B	1.0927 (5)	0.1196 (5)	0.0574 (3)	0.0387 (10)	0.5
H64B	1.097031	0.082896	0.023361	0.046*	0.5
C65B	1.1761 (3)	0.1065 (3)	0.10415 (19)	0.0461 (8)	0.5
H65B	1.237395	0.060883	0.101487	0.055*	0.5
C66B	1.1697 (4)	0.1592 (3)	0.15362 (19)	0.0465 (9)	0.5
H66B	1.226463	0.149678	0.184864	0.056*	0.5
C67B	1.0823 (4)	0.2252 (4)	0.1581 (3)	0.0465 (11)	0.5
H67B	1.077876	0.261616	0.192212	0.056*	0.5
C68B	0.9993 (4)	0.2384 (4)	0.1118 (2)	0.0457 (10)	0.5
H68B	0.938143	0.283953	0.115108	0.055*	0.5
C69B	0.9157 (6)	0.2000 (7)	0.0132 (3)	0.064 (2)	0.5
H69D	0.854614	0.155444	0.030432	0.096*	0.5
H69E	0.944982	0.181047	−0.026316	0.096*	0.5
H69F	0.888968	0.272002	0.003462	0.096*	0.5
C70	0.4917 (5)	1.0253 (4)	−0.0179 (2)	0.0336 (11)*	0.290 (3)
C71	0.5723 (6)	0.9686 (5)	0.0206 (3)	0.0257 (10)*	0.290 (3)
H71	0.647787	0.975667	0.008032	0.031*	0.290 (3)
C72	0.5438 (4)	0.9018 (4)	0.0773 (3)	0.0294 (10)*	0.290 (3)
H72	0.599320	0.862207	0.103469	0.035*	0.290 (3)
C73	0.4321 (5)	0.8927 (4)	0.0962 (3)	0.0321 (11)*	0.290 (3)
H73	0.411131	0.847813	0.135316	0.038*	0.290 (3)
C74	0.3548 (6)	0.9494 (5)	0.0572 (3)	0.0442 (14)*	0.290 (3)
H74	0.279015	0.941405	0.068909	0.053*	0.290 (3)
C75	0.3825 (6)	1.0179 (6)	0.0014 (4)	0.0313 (14)*	0.290 (3)
H75	0.326996	1.059707	−0.023554	0.038*	0.290 (3)
C76	0.5240 (7)	1.0995 (5)	−0.0797 (3)	0.0466 (15)*	0.290 (3)
H76A	0.577987	1.065504	−0.104025	0.070*	0.290 (3)
H76B	0.556529	1.160294	−0.069018	0.070*	0.290 (3)
H76C	0.458205	1.120795	−0.106054	0.070*	0.290 (3)
C70B	0.5530 (4)	1.0402 (4)	−0.0334 (2)	0.0349 (16)*	0.210 (3)
C71B	0.5971 (4)	0.9610 (5)	0.0139 (3)	0.0243 (17)*	0.210 (3)
H71B	0.674628	0.947487	0.015495	0.029*	0.210 (3)
C72B	0.5278 (6)	0.9016 (4)	0.0588 (3)	0.055 (3)*	0.210 (3)
H72B	0.557933	0.847531	0.091086	0.066*	0.210 (3)
C73B	0.4143 (6)	0.9214 (6)	0.0564 (3)	0.070 (3)*	0.210 (3)
H73B	0.366959	0.880824	0.087125	0.084*	0.210 (3)
C74B	0.3702 (3)	1.0006 (6)	0.0092 (4)	0.054 (4)*	0.210 (3)
H74B	0.292679	1.014073	0.007573	0.065*	0.210 (3)
C75B	0.4395 (4)	1.0599 (5)	−0.0357 (3)	0.0337 (17)*	0.210 (3)
H75B	0.409373	1.114031	−0.068019	0.040*	0.210 (3)
C76B	0.6380 (13)	1.0964 (13)	−0.0841 (8)	0.084 (4)*	0.210 (3)
H76D	0.695364	1.125248	−0.061800	0.127*	0.210 (3)
H76E	0.599874	1.151991	−0.114432	0.127*	0.210 (3)
H76F	0.671931	1.047048	−0.107786	0.127*	0.210 (3)

### Tris(dicyclohexylamido)(4-fluorobenzonitrile)titanium methyltris(pentafluorophenyl)borate toluene sesquisolvate (2) Atomic displacement parameters (Å^2^)

**Table:**

	*U*^11^	*U*^22^	*U*^33^	*U*^12^	*U*^13^	*U*^23^
Ti1	0.01124 (7)	0.01433 (8)	0.01625 (8)	−0.00037 (6)	−0.00221 (6)	0.00233 (6)
F1	0.0394 (4)	0.0171 (3)	0.0286 (4)	−0.0027 (3)	−0.0026 (3)	0.0066 (3)
N1	0.0154 (4)	0.0135 (4)	0.0297 (5)	−0.0002 (3)	−0.0050 (3)	−0.0026 (3)
N2	0.0131 (3)	0.0170 (4)	0.0151 (4)	−0.0010 (3)	−0.0024 (3)	0.0006 (3)
N3	0.0124 (3)	0.0200 (4)	0.0203 (4)	−0.0008 (3)	−0.0029 (3)	0.0008 (3)
N4	0.0168 (4)	0.0180 (4)	0.0188 (4)	0.0014 (3)	−0.0013 (3)	−0.0003 (3)
C1	0.0177 (4)	0.0174 (4)	0.0200 (4)	0.0024 (3)	−0.0043 (3)	−0.0088 (4)
C2	0.0214 (5)	0.0284 (6)	0.0240 (5)	0.0083 (4)	−0.0025 (4)	−0.0109 (4)
C3	0.0225 (5)	0.0397 (7)	0.0262 (6)	0.0037 (5)	0.0008 (4)	−0.0154 (5)
C4	0.0328 (6)	0.0386 (7)	0.0228 (5)	−0.0024 (5)	0.0034 (5)	−0.0124 (5)
C5	0.0347 (6)	0.0264 (6)	0.0190 (5)	−0.0013 (5)	−0.0050 (4)	−0.0086 (4)
C6	0.0254 (5)	0.0200 (5)	0.0214 (5)	0.0021 (4)	−0.0090 (4)	−0.0086 (4)
C7A	0.0158 (5)	0.0132 (5)	0.0179 (6)	−0.0011 (4)	0.0003 (4)	−0.0050 (4)
C8A	0.0178 (6)	0.0195 (6)	0.0170 (6)	−0.0037 (5)	−0.0012 (5)	−0.0043 (5)
C9A	0.0224 (6)	0.0253 (7)	0.0226 (7)	−0.0092 (5)	0.0035 (5)	−0.0112 (6)
C10A	0.0254 (7)	0.0198 (7)	0.0270 (7)	−0.0072 (6)	0.0053 (6)	−0.0072 (6)
C11A	0.0292 (8)	0.0161 (6)	0.0323 (8)	−0.0018 (5)	−0.0025 (6)	−0.0042 (6)
C12A	0.0272 (7)	0.0153 (6)	0.0220 (7)	−0.0019 (5)	−0.0054 (5)	−0.0012 (5)
C13	0.0125 (4)	0.0150 (4)	0.0152 (4)	0.0004 (3)	−0.0010 (3)	−0.0021 (3)
C14	0.0169 (4)	0.0147 (4)	0.0209 (5)	0.0021 (3)	−0.0026 (3)	−0.0036 (4)
C15	0.0212 (5)	0.0170 (5)	0.0243 (5)	0.0026 (4)	−0.0066 (4)	−0.0008 (4)
C16	0.0199 (5)	0.0246 (5)	0.0292 (6)	−0.0001 (4)	−0.0104 (4)	0.0002 (4)
C17	0.0284 (5)	0.0201 (5)	0.0247 (5)	−0.0040 (4)	−0.0117 (4)	−0.0025 (4)
C18	0.0175 (4)	0.0151 (4)	0.0189 (4)	−0.0013 (3)	−0.0040 (3)	−0.0023 (3)
C19	0.0179 (4)	0.0205 (5)	0.0136 (4)	−0.0050 (4)	−0.0012 (3)	−0.0008 (3)
C20	0.0247 (5)	0.0175 (5)	0.0159 (4)	0.0010 (4)	−0.0021 (4)	−0.0014 (4)
C21	0.0383 (6)	0.0212 (5)	0.0176 (5)	−0.0034 (5)	−0.0020 (4)	0.0012 (4)
C22	0.0318 (6)	0.0356 (7)	0.0157 (5)	−0.0019 (5)	0.0002 (4)	−0.0027 (4)
C23	0.0250 (6)	0.0511 (8)	0.0196 (5)	0.0011 (5)	0.0040 (4)	−0.0043 (5)
C24	0.0201 (5)	0.0335 (6)	0.0189 (5)	0.0054 (4)	−0.0001 (4)	−0.0034 (4)
C25	0.0158 (4)	0.0202 (5)	0.0199 (5)	−0.0029 (3)	−0.0030 (3)	−0.0011 (4)
C26	0.0288 (6)	0.0254 (6)	0.0220 (5)	−0.0028 (4)	−0.0034 (4)	−0.0041 (4)
C27	0.0416 (7)	0.0346 (7)	0.0297 (6)	0.0036 (6)	0.0005 (5)	−0.0106 (5)
C28	0.0515 (9)	0.0261 (7)	0.0459 (8)	0.0018 (6)	−0.0035 (7)	−0.0137 (6)
C29	0.0379 (7)	0.0212 (6)	0.0421 (8)	0.0004 (5)	−0.0001 (6)	−0.0023 (5)
C30	0.0198 (5)	0.0203 (5)	0.0308 (6)	−0.0042 (4)	−0.0020 (4)	−0.0002 (4)
C31	0.0131 (4)	0.0346 (6)	0.0207 (5)	0.0023 (4)	−0.0039 (4)	−0.0011 (4)
C32A	0.0173 (6)	0.0287 (8)	0.0685 (13)	0.0026 (5)	−0.0047 (7)	0.0140 (8)
C33A	0.0191 (7)	0.0564 (13)	0.0720 (16)	0.0111 (7)	−0.0030 (8)	0.0301 (12)
C34A	0.0165 (6)	0.0445 (10)	0.0499 (10)	0.0080 (6)	−0.0056 (6)	−0.0035 (8)
C35A	0.0125 (5)	0.0508 (10)	0.0389 (8)	0.0015 (6)	−0.0016 (5)	−0.0188 (7)
C36A	0.0140 (5)	0.0274 (7)	0.0373 (8)	−0.0007 (5)	0.0014 (5)	−0.0096 (6)
C37	0.0159 (4)	0.0184 (5)	0.0181 (4)	0.0008 (3)	−0.0021 (3)	−0.0024 (4)
C38	0.0170 (4)	0.0140 (4)	0.0165 (4)	0.0008 (3)	−0.0019 (3)	−0.0002 (3)
C39	0.0167 (4)	0.0206 (5)	0.0226 (5)	−0.0014 (4)	−0.0048 (4)	−0.0008 (4)
C40	0.0205 (5)	0.0180 (5)	0.0237 (5)	−0.0047 (4)	−0.0010 (4)	−0.0023 (4)
C41	0.0256 (5)	0.0137 (4)	0.0187 (5)	0.0003 (4)	0.0004 (4)	0.0008 (4)
C42	0.0231 (5)	0.0212 (5)	0.0295 (6)	−0.0010 (4)	−0.0094 (4)	0.0053 (4)
C43	0.0194 (5)	0.0191 (5)	0.0282 (5)	−0.0030 (4)	−0.0076 (4)	0.0042 (4)
F2	0.0200 (3)	0.0257 (4)	0.0325 (4)	−0.0058 (3)	−0.0057 (3)	−0.0031 (3)
F3	0.0392 (4)	0.0237 (4)	0.0318 (4)	−0.0086 (3)	0.0075 (3)	−0.0055 (3)
F4	0.0606 (6)	0.0280 (4)	0.0226 (4)	0.0073 (4)	−0.0036 (4)	−0.0006 (3)
F5	0.0411 (5)	0.0377 (5)	0.0434 (5)	0.0115 (4)	−0.0253 (4)	−0.0102 (4)
F6	0.0195 (3)	0.0321 (4)	0.0485 (5)	−0.0027 (3)	−0.0116 (3)	−0.0087 (4)
F7	0.0169 (3)	0.0217 (3)	0.0262 (3)	−0.0035 (2)	0.0023 (2)	−0.0057 (3)
F8	0.0255 (3)	0.0300 (4)	0.0236 (3)	0.0070 (3)	0.0002 (3)	−0.0097 (3)
F9	0.0420 (4)	0.0208 (3)	0.0325 (4)	0.0045 (3)	−0.0126 (3)	−0.0136 (3)
F10	0.0327 (4)	0.0225 (3)	0.0356 (4)	−0.0128 (3)	−0.0089 (3)	−0.0039 (3)
F11	0.0210 (3)	0.0335 (4)	0.0270 (3)	−0.0115 (3)	0.0040 (3)	−0.0075 (3)
F12	0.0328 (4)	0.0231 (3)	0.0286 (4)	0.0052 (3)	−0.0073 (3)	−0.0067 (3)
F13	0.0340 (4)	0.0447 (5)	0.0370 (5)	0.0084 (4)	−0.0132 (4)	0.0035 (4)
F14	0.0454 (5)	0.0627 (6)	0.0306 (4)	−0.0176 (5)	−0.0200 (4)	−0.0006 (4)
F15	0.0687 (6)	0.0400 (5)	0.0323 (4)	−0.0210 (5)	−0.0113 (4)	−0.0150 (4)
F16	0.0472 (5)	0.0240 (4)	0.0360 (4)	0.0005 (3)	−0.0056 (4)	−0.0154 (3)
C44	0.0169 (4)	0.0168 (4)	0.0247 (5)	0.0015 (3)	−0.0027 (4)	−0.0078 (4)
C45	0.0194 (4)	0.0184 (5)	0.0241 (5)	0.0010 (4)	−0.0030 (4)	−0.0078 (4)
C46	0.0294 (5)	0.0179 (5)	0.0242 (5)	−0.0004 (4)	0.0019 (4)	−0.0083 (4)
C47	0.0392 (6)	0.0193 (5)	0.0211 (5)	0.0071 (5)	−0.0033 (5)	−0.0062 (4)
C48	0.0303 (6)	0.0244 (6)	0.0293 (6)	0.0095 (5)	−0.0117 (5)	−0.0110 (5)
C49	0.0197 (5)	0.0206 (5)	0.0313 (6)	0.0019 (4)	−0.0058 (4)	−0.0094 (4)
C50	0.0161 (4)	0.0171 (4)	0.0189 (4)	−0.0019 (3)	−0.0017 (3)	−0.0039 (4)
C51	0.0165 (4)	0.0161 (4)	0.0193 (4)	−0.0003 (3)	−0.0025 (3)	−0.0026 (4)
C52	0.0209 (4)	0.0183 (5)	0.0178 (4)	0.0057 (4)	−0.0039 (4)	−0.0037 (4)
C53	0.0284 (5)	0.0147 (4)	0.0220 (5)	0.0037 (4)	−0.0107 (4)	−0.0054 (4)
C54	0.0237 (5)	0.0154 (4)	0.0234 (5)	−0.0041 (4)	−0.0085 (4)	−0.0009 (4)
C55	0.0178 (4)	0.0203 (5)	0.0195 (5)	−0.0037 (4)	−0.0024 (4)	−0.0030 (4)
C56	0.0215 (5)	0.0197 (5)	0.0189 (5)	−0.0040 (4)	−0.0004 (4)	−0.0045 (4)
C57	0.0228 (5)	0.0222 (5)	0.0206 (5)	−0.0031 (4)	−0.0017 (4)	−0.0028 (4)
C58	0.0249 (5)	0.0315 (6)	0.0241 (5)	−0.0028 (5)	−0.0048 (4)	0.0030 (5)
C59	0.0323 (6)	0.0420 (8)	0.0210 (5)	−0.0156 (5)	−0.0087 (5)	0.0007 (5)
C60	0.0408 (7)	0.0317 (6)	0.0226 (5)	−0.0163 (5)	−0.0035 (5)	−0.0076 (5)
C61	0.0316 (6)	0.0232 (5)	0.0229 (5)	−0.0056 (4)	−0.0024 (4)	−0.0065 (4)
C62	0.0237 (5)	0.0361 (7)	0.0363 (7)	−0.0040 (5)	0.0075 (5)	−0.0168 (6)
B1	0.0167 (5)	0.0191 (5)	0.0230 (5)	−0.0021 (4)	−0.0004 (4)	−0.0076 (4)
C63A	0.0316 (13)	0.0357 (15)	0.0337 (14)	−0.0109 (11)	−0.0015 (11)	0.0087 (11)
C64A	0.030 (2)	0.072 (3)	0.056 (4)	0.015 (2)	−0.005 (2)	−0.008 (3)
C65A	0.062 (3)	0.092 (4)	0.058 (3)	0.008 (2)	0.006 (2)	−0.039 (3)
C66A	0.051 (2)	0.077 (3)	0.0391 (19)	−0.013 (2)	0.0009 (17)	−0.008 (2)
C67A	0.042 (2)	0.075 (4)	0.048 (3)	0.013 (2)	−0.002 (3)	0.005 (3)
C68A	0.057 (3)	0.040 (2)	0.044 (3)	0.0140 (19)	−0.0040 (19)	−0.0051 (19)
C69A	0.047 (3)	0.056 (3)	0.057 (3)	−0.016 (3)	−0.006 (3)	0.010 (2)
C63B	0.0305 (13)	0.0408 (16)	0.0405 (16)	−0.0032 (12)	0.0004 (12)	0.0106 (13)
C64B	0.032 (2)	0.0411 (19)	0.040 (3)	0.0056 (16)	−0.0020 (17)	−0.003 (2)
C65B	0.0390 (17)	0.0400 (17)	0.053 (2)	0.0068 (13)	−0.0040 (15)	0.0037 (15)
C66B	0.048 (2)	0.043 (2)	0.0397 (18)	−0.0163 (16)	−0.0117 (15)	0.0134 (15)
C67B	0.063 (3)	0.033 (2)	0.044 (3)	−0.015 (2)	0.021 (2)	−0.0081 (17)
C68B	0.052 (3)	0.0318 (17)	0.050 (3)	−0.0054 (19)	0.017 (2)	−0.002 (2)
C69B	0.044 (3)	0.077 (5)	0.059 (4)	−0.004 (3)	−0.009 (3)	0.018 (4)

### Tris(dicyclohexylamido)(4-fluorobenzonitrile)titanium methyltris(pentafluorophenyl)borate toluene sesquisolvate (2) Geometric parameters (Å, º)

**Table:**

Ti1—N2	1.8869 (9)	C34A—H34B	0.9900
Ti1—N1	1.8912 (10)	C35A—C36A	1.525 (2)
Ti1—N3	1.8921 (9)	C35A—H35A	0.9900
Ti1—N4	2.1608 (9)	C35A—H35B	0.9900
Ti1—C13	2.6187 (10)	C36A—H36A	0.9900
Ti1—C25	2.6338 (11)	C36A—H36B	0.9900
Ti1—C1	2.6802 (11)	C32B—C33B	1.533 (13)
F1—C41	1.3465 (12)	C32B—H32C	0.9900
N1—C1	1.4802 (15)	C32B—H32D	0.9900
N1—C7A	1.4966 (16)	C33B—C34B	1.468 (16)
N1—C7B	1.503 (5)	C33B—H33C	0.9900
N2—C19	1.4714 (13)	C33B—H33D	0.9900
N2—C13	1.4817 (13)	C34B—C35B	1.541 (15)
N3—C31	1.4721 (13)	C34B—H34C	0.9900
N3—C25	1.4859 (15)	C34B—H34D	0.9900
N4—C37	1.1472 (14)	C35B—C36B	1.561 (14)
C1—C2	1.5370 (15)	C35B—H35C	0.9900
C1—C6	1.5407 (15)	C35B—H35D	0.9900
C1—H1	1.0000	C36B—H36C	0.9900
C2—C3	1.5311 (19)	C36B—H36D	0.9900
C2—H2A	0.9900	C37—C38	1.4282 (14)
C2—H2B	0.9900	C38—C43	1.3949 (14)
C3—C4	1.5325 (19)	C38—C39	1.3964 (15)
C3—H3A	0.9900	C39—C40	1.3828 (15)
C3—H3B	0.9900	C39—H39	0.9500
C4—C5	1.5288 (19)	C40—C41	1.3784 (16)
C4—H4A	0.9900	C40—H40	0.9500
C4—H4B	0.9900	C41—C42	1.3810 (16)
C5—C6	1.5252 (18)	C42—C43	1.3818 (16)
C5—H5A	0.9900	C42—H42	0.9500
C5—H5B	0.9900	C43—H43	0.9500
C6—H6A	0.9900	F2—C45	1.3464 (13)
C6—H6B	0.9900	F3—C46	1.3402 (14)
C7A—C8A	1.5315 (19)	F4—C47	1.3417 (14)
C7A—C12A	1.532 (2)	F5—C48	1.3527 (14)
C7A—H7A	1.0000	F6—C49	1.3598 (14)
C8A—C9A	1.529 (2)	F7—C51	1.3499 (12)
C8A—H8AA	0.9900	F8—C52	1.3489 (13)
C8A—H8AB	0.9900	F9—C53	1.3386 (13)
C9A—C10A	1.528 (2)	F10—C54	1.3485 (13)
C9A—H9AA	0.9900	F11—C55	1.3571 (13)
C9A—H9AB	0.9900	F12—C57	1.3559 (14)
C10A—C11A	1.527 (2)	F13—C58	1.3432 (16)
C10A—H10A	0.9900	F14—C59	1.3450 (14)
C10A—H10B	0.9900	F15—C60	1.3488 (16)
C11A—C12A	1.531 (2)	F16—C61	1.3506 (15)
C11A—H11A	0.9900	C44—C45	1.3912 (16)
C11A—H11B	0.9900	C44—C49	1.3941 (15)
C12A—H12A	0.9900	C44—B1	1.6625 (17)
C12A—H12B	0.9900	C45—C46	1.3887 (17)
C7B—C12B	1.516 (7)	C46—C47	1.3788 (18)
C7B—C8B	1.523 (7)	C47—C48	1.372 (2)
C7B—H7B	1.0000	C48—C49	1.3801 (18)
C8B—C9B	1.555 (8)	C50—C55	1.3873 (15)
C8B—H8BA	0.9900	C50—C51	1.3978 (15)
C8B—H8BB	0.9900	C50—B1	1.6594 (16)
C9B—C10B	1.526 (8)	C51—C52	1.3796 (15)
C9B—H9BA	0.9900	C52—C53	1.3835 (16)
C9B—H9BB	0.9900	C53—C54	1.3759 (18)
C10B—C11B	1.505 (9)	C54—C55	1.3865 (16)
C10B—H10C	0.9900	C56—C57	1.3891 (16)
C10B—H10D	0.9900	C56—C61	1.3977 (16)
C11B—C12B	1.529 (8)	C56—B1	1.6573 (16)
C11B—H11C	0.9900	C57—C58	1.3890 (16)
C11B—H11D	0.9900	C58—C59	1.374 (2)
C12B—H12C	0.9900	C59—C60	1.376 (2)
C12B—H12D	0.9900	C60—C61	1.3825 (18)
C13—C18	1.5337 (14)	C62—B1	1.6417 (17)
C13—C14	1.5399 (14)	C62—H62A	0.9800
C13—H13	1.0000	C62—H62B	0.9800
C14—C15	1.5312 (15)	C62—H62C	0.9800
C14—H14A	0.9900	C63A—C68A	1.372 (6)
C14—H14B	0.9900	C63A—C64A	1.389 (9)
C15—C16	1.5271 (16)	C63A—C69A	1.483 (7)
C15—H15A	0.9900	C64A—C65A	1.399 (7)
C15—H15B	0.9900	C64A—H64A	0.9500
C16—C17	1.5280 (17)	C65A—C66A	1.365 (7)
C16—H16A	0.9900	C65A—H65A	0.9500
C16—H16B	0.9900	C66A—C67A	1.391 (9)
C17—C18	1.5326 (15)	C66A—H66A	0.9500
C17—H17A	0.9900	C67A—C68A	1.361 (8)
C17—H17B	0.9900	C67A—H67A	0.9500
C18—H18A	0.9900	C68A—H68A	0.9500
C18—H18B	0.9900	C69A—H69A	0.9800
C19—C20	1.5258 (15)	C69A—H69B	0.9800
C19—C24	1.5350 (16)	C69A—H69C	0.9800
C19—H19	1.0000	C63B—C68B	1.375 (6)
C20—C21	1.5299 (16)	C63B—C64B	1.388 (7)
C20—H20A	0.9900	C63B—C69B	1.479 (8)
C20—H20B	0.9900	C64B—C65B	1.410 (6)
C21—C22	1.5225 (19)	C64B—H64B	0.9500
C21—H21A	0.9900	C65B—C66B	1.375 (6)
C21—H21B	0.9900	C65B—H65B	0.9500
C22—C23	1.5205 (19)	C66B—C67B	1.365 (6)
C22—H22A	0.9900	C66B—H66B	0.9500
C22—H22B	0.9900	C67B—C68B	1.398 (8)
C23—C24	1.5369 (18)	C67B—H67B	0.9500
C23—H23A	0.9900	C68B—H68B	0.9500
C23—H23B	0.9900	C69B—H69D	0.9800
C24—H24A	0.9900	C69B—H69E	0.9800
C24—H24B	0.9900	C69B—H69F	0.9800
C25—C30	1.5367 (16)	C70—C75	1.378 (10)
C25—C26	1.5371 (16)	C70—C71	1.381 (8)
C25—H25	1.0000	C70—C76	1.518 (9)
C26—C27	1.5307 (19)	C71—C72	1.382 (8)
C26—H26A	0.9900	C71—H71	0.9500
C26—H26B	0.9900	C72—C73	1.405 (7)
C27—C28	1.522 (2)	C72—H72	0.9500
C27—H27A	0.9900	C73—C74	1.358 (9)
C27—H27B	0.9900	C73—H73	0.9500
C28—C29	1.528 (2)	C74—C75	1.376 (11)
C28—H28A	0.9900	C74—H74	0.9500
C28—H28B	0.9900	C75—H75	0.9500
C29—C30	1.5287 (19)	C76—H76A	0.9800
C29—H29A	0.9900	C76—H76B	0.9800
C29—H29B	0.9900	C76—H76C	0.9800
C30—H30A	0.9900	C70B—C71B	1.3900
C30—H30B	0.9900	C70B—C75B	1.3900
C31—C36B	1.416 (10)	C70B—C76B	1.564 (16)
C31—C32B	1.504 (8)	C71B—C72B	1.3900
C31—C36A	1.5332 (18)	C71B—H71B	0.9500
C31—C32A	1.535 (2)	C72B—C73B	1.3900
C31—H31A	1.0000	C72B—H72B	0.9500
C31—H31	1.0000	C73B—C74B	1.3900
C32A—C33A	1.532 (2)	C73B—H73B	0.9500
C32A—H32A	0.9900	C74B—C75B	1.3900
C32A—H32B	0.9900	C74B—H74B	0.9500
C33A—C34A	1.527 (3)	C75B—H75B	0.9500
C33A—H33A	0.9900	C76B—H76D	0.9800
C33A—H33B	0.9900	C76B—H76E	0.9800
C34A—C35A	1.507 (3)	C76B—H76F	0.9800
C34A—H34A	0.9900		
			
N2—Ti1—N1	109.82 (4)	C25—C30—H30A	109.6
N2—Ti1—N3	111.51 (4)	C29—C30—H30B	109.6
N1—Ti1—N3	113.62 (4)	C25—C30—H30B	109.6
N2—Ti1—N4	111.16 (4)	H30A—C30—H30B	108.1
N1—Ti1—N4	105.83 (4)	C36B—C31—N3	110.4 (4)
N3—Ti1—N4	104.66 (4)	C36B—C31—C32B	114.4 (6)
N2—Ti1—C13	33.69 (3)	N3—C31—C32B	113.4 (3)
N1—Ti1—C13	99.61 (4)	N3—C31—C36A	114.10 (10)
N3—Ti1—C13	140.91 (4)	N3—C31—C32A	110.87 (10)
N4—Ti1—C13	84.34 (3)	C36A—C31—C32A	108.18 (12)
N2—Ti1—C25	98.25 (4)	C36B—C31—H31A	106.0
N1—Ti1—C25	145.04 (4)	N3—C31—H31A	106.0
N3—Ti1—C25	33.52 (4)	C32B—C31—H31A	106.0
N4—Ti1—C25	81.84 (4)	N3—C31—H31	107.8
C13—Ti1—C25	115.20 (3)	C36A—C31—H31	107.8
N2—Ti1—C1	140.19 (4)	C32A—C31—H31	107.8
N1—Ti1—C1	32.30 (4)	C33A—C32A—C31	111.24 (16)
N3—Ti1—C1	100.32 (4)	C33A—C32A—H32A	109.4
N4—Ti1—C1	81.74 (3)	C31—C32A—H32A	109.4
C13—Ti1—C1	118.70 (3)	C33A—C32A—H32B	109.4
C25—Ti1—C1	121.21 (3)	C31—C32A—H32B	109.4
C1—N1—C7A	111.84 (9)	H32A—C32A—H32B	108.0
C1—N1—C7B	139.1 (2)	C34A—C33A—C32A	111.38 (15)
C1—N1—Ti1	104.65 (6)	C34A—C33A—H33A	109.4
C7A—N1—Ti1	142.82 (8)	C32A—C33A—H33A	109.4
C7B—N1—Ti1	116.1 (2)	C34A—C33A—H33B	109.4
C19—N2—C13	118.17 (8)	C32A—C33A—H33B	109.4
C19—N2—Ti1	140.35 (7)	H33A—C33A—H33B	108.0
C13—N2—Ti1	101.37 (6)	C35A—C34A—C33A	111.25 (15)
C31—N3—C25	115.66 (9)	C35A—C34A—H34A	109.4
C31—N3—Ti1	142.51 (8)	C33A—C34A—H34A	109.4
C25—N3—Ti1	101.79 (6)	C35A—C34A—H34B	109.4
C37—N4—Ti1	175.04 (9)	C33A—C34A—H34B	109.4
N1—C1—C2	115.83 (9)	H34A—C34A—H34B	108.0
N1—C1—C6	113.66 (9)	C34A—C35A—C36A	111.70 (13)
C2—C1—C6	109.26 (9)	C34A—C35A—H35A	109.3
N1—C1—Ti1	43.05 (5)	C36A—C35A—H35A	109.3
C2—C1—Ti1	135.39 (7)	C34A—C35A—H35B	109.3
C6—C1—Ti1	115.33 (7)	C36A—C35A—H35B	109.3
N1—C1—H1	105.7	H35A—C35A—H35B	107.9
C2—C1—H1	105.7	C35A—C36A—C31	110.73 (13)
C6—C1—H1	105.7	C35A—C36A—H36A	109.5
Ti1—C1—H1	63.8	C31—C36A—H36A	109.5
C3—C2—C1	110.16 (10)	C35A—C36A—H36B	109.5
C3—C2—H2A	109.6	C31—C36A—H36B	109.5
C1—C2—H2A	109.6	H36A—C36A—H36B	108.1
C3—C2—H2B	109.6	C31—C32B—C33B	111.7 (7)
C1—C2—H2B	109.6	C31—C32B—H32C	109.3
H2A—C2—H2B	108.1	C33B—C32B—H32C	109.3
C2—C3—C4	112.01 (11)	C31—C32B—H32D	109.3
C2—C3—H3A	109.2	C33B—C32B—H32D	109.3
C4—C3—H3A	109.2	H32C—C32B—H32D	107.9
C2—C3—H3B	109.2	C34B—C33B—C32B	109.1 (9)
C4—C3—H3B	109.2	C34B—C33B—H33C	109.9
H3A—C3—H3B	107.9	C32B—C33B—H33C	109.9
C5—C4—C3	110.85 (10)	C34B—C33B—H33D	109.9
C5—C4—H4A	109.5	C32B—C33B—H33D	109.9
C3—C4—H4A	109.5	H33C—C33B—H33D	108.3
C5—C4—H4B	109.5	C33B—C34B—C35B	112.0 (9)
C3—C4—H4B	109.5	C33B—C34B—H34C	109.2
H4A—C4—H4B	108.1	C35B—C34B—H34C	109.2
C6—C5—C4	110.75 (11)	C33B—C34B—H34D	109.2
C6—C5—H5A	109.5	C35B—C34B—H34D	109.2
C4—C5—H5A	109.5	H34C—C34B—H34D	107.9
C6—C5—H5B	109.5	C34B—C35B—C36B	108.3 (8)
C4—C5—H5B	109.5	C34B—C35B—H35C	110.0
H5A—C5—H5B	108.1	C36B—C35B—H35C	110.0
C5—C6—C1	110.97 (9)	C34B—C35B—H35D	110.0
C5—C6—H6A	109.4	C36B—C35B—H35D	110.0
C1—C6—H6A	109.4	H35C—C35B—H35D	108.4
C5—C6—H6B	109.4	C31—C36B—C35B	114.2 (8)
C1—C6—H6B	109.4	C31—C36B—H36C	108.7
H6A—C6—H6B	108.0	C35B—C36B—H36C	108.7
N1—C7A—C8A	114.54 (11)	C31—C36B—H36D	108.7
N1—C7A—C12A	109.42 (11)	C35B—C36B—H36D	108.7
C8A—C7A—C12A	109.50 (11)	H36C—C36B—H36D	107.6
N1—C7A—H7A	107.7	N4—C37—C38	179.25 (12)
C8A—C7A—H7A	107.7	C43—C38—C39	121.35 (10)
C12A—C7A—H7A	107.7	C43—C38—C37	119.19 (10)
C9A—C8A—C7A	109.98 (12)	C39—C38—C37	119.45 (9)
C9A—C8A—H8AA	109.7	C40—C39—C38	119.14 (10)
C7A—C8A—H8AA	109.7	C40—C39—H39	120.4
C9A—C8A—H8AB	109.7	C38—C39—H39	120.4
C7A—C8A—H8AB	109.7	C41—C40—C39	118.37 (10)
H8AA—C8A—H8AB	108.2	C41—C40—H40	120.8
C10A—C9A—C8A	110.97 (12)	C39—C40—H40	120.8
C10A—C9A—H9AA	109.4	F1—C41—C40	118.22 (10)
C8A—C9A—H9AA	109.4	F1—C41—C42	118.18 (10)
C10A—C9A—H9AB	109.4	C40—C41—C42	123.59 (10)
C8A—C9A—H9AB	109.4	C41—C42—C43	118.11 (10)
H9AA—C9A—H9AB	108.0	C41—C42—H42	120.9
C11A—C10A—C9A	111.41 (13)	C43—C42—H42	120.9
C11A—C10A—H10A	109.3	C42—C43—C38	119.42 (10)
C9A—C10A—H10A	109.3	C42—C43—H43	120.3
C11A—C10A—H10B	109.3	C38—C43—H43	120.3
C9A—C10A—H10B	109.3	C45—C44—C49	112.78 (10)
H10A—C10A—H10B	108.0	C45—C44—B1	127.51 (9)
C10A—C11A—C12A	111.50 (13)	C49—C44—B1	119.56 (10)
C10A—C11A—H11A	109.3	F2—C45—C46	115.02 (10)
C12A—C11A—H11A	109.3	F2—C45—C44	120.52 (10)
C10A—C11A—H11B	109.3	C46—C45—C44	124.44 (11)
C12A—C11A—H11B	109.3	F3—C46—C47	119.74 (11)
H11A—C11A—H11B	108.0	F3—C46—C45	120.76 (11)
C11A—C12A—C7A	110.85 (13)	C47—C46—C45	119.50 (11)
C11A—C12A—H12A	109.5	F4—C47—C48	120.41 (12)
C7A—C12A—H12A	109.5	F4—C47—C46	120.76 (12)
C11A—C12A—H12B	109.5	C48—C47—C46	118.83 (11)
C7A—C12A—H12B	109.5	F5—C48—C47	119.69 (12)
H12A—C12A—H12B	108.1	F5—C48—C49	120.62 (12)
N1—C7B—C12B	114.3 (3)	C47—C48—C49	119.69 (11)
N1—C7B—C8B	106.5 (4)	F6—C49—C48	115.25 (10)
C12B—C7B—C8B	111.7 (4)	F6—C49—C44	119.96 (11)
N1—C7B—H7B	108.1	C48—C49—C44	124.77 (11)
C12B—C7B—H7B	108.1	C55—C50—C51	112.99 (10)
C8B—C7B—H7B	108.1	C55—C50—B1	126.72 (10)
C7B—C8B—C9B	109.9 (4)	C51—C50—B1	120.19 (9)
C7B—C8B—H8BA	109.7	F7—C51—C52	115.88 (9)
C9B—C8B—H8BA	109.7	F7—C51—C50	119.38 (9)
C7B—C8B—H8BB	109.7	C52—C51—C50	124.70 (10)
C9B—C8B—H8BB	109.7	F8—C52—C51	120.94 (10)
H8BA—C8B—H8BB	108.2	F8—C52—C53	119.55 (10)
C10B—C9B—C8B	109.6 (5)	C51—C52—C53	119.50 (10)
C10B—C9B—H9BA	109.7	F9—C53—C54	121.23 (11)
C8B—C9B—H9BA	109.7	F9—C53—C52	120.31 (11)
C10B—C9B—H9BB	109.7	C54—C53—C52	118.46 (10)
C8B—C9B—H9BB	109.7	F10—C54—C53	120.20 (10)
H9BA—C9B—H9BB	108.2	F10—C54—C55	119.79 (11)
C11B—C10B—C9B	113.4 (6)	C53—C54—C55	120.01 (10)
C11B—C10B—H10C	108.9	F11—C55—C54	114.41 (9)
C9B—C10B—H10C	108.9	F11—C55—C50	121.28 (10)
C11B—C10B—H10D	108.9	C54—C55—C50	124.31 (11)
C9B—C10B—H10D	108.9	C57—C56—C61	112.63 (10)
H10C—C10B—H10D	107.7	C57—C56—B1	127.64 (10)
C10B—C11B—C12B	109.9 (5)	C61—C56—B1	119.28 (10)
C10B—C11B—H11C	109.7	F12—C57—C58	113.96 (11)
C12B—C11B—H11C	109.7	F12—C57—C56	121.40 (10)
C10B—C11B—H11D	109.7	C58—C57—C56	124.64 (12)
C12B—C11B—H11D	109.7	F13—C58—C59	120.15 (12)
H11C—C11B—H11D	108.2	F13—C58—C57	120.26 (12)
C7B—C12B—C11B	110.1 (4)	C59—C58—C57	119.59 (12)
C7B—C12B—H12C	109.6	F14—C59—C58	120.54 (14)
C11B—C12B—H12C	109.6	F14—C59—C60	120.61 (13)
C7B—C12B—H12D	109.6	C58—C59—C60	118.84 (11)
C11B—C12B—H12D	109.6	F15—C60—C59	119.57 (12)
H12C—C12B—H12D	108.1	F15—C60—C61	120.88 (13)
N2—C13—C18	115.08 (8)	C59—C60—C61	119.55 (12)
N2—C13—C14	115.14 (8)	F16—C61—C60	115.54 (11)
C18—C13—C14	109.08 (8)	F16—C61—C56	119.76 (11)
N2—C13—Ti1	44.94 (4)	C60—C61—C56	124.69 (12)
C18—C13—Ti1	139.35 (7)	B1—C62—H62A	109.5
C14—C13—Ti1	111.57 (6)	B1—C62—H62B	109.5
N2—C13—H13	105.5	H62A—C62—H62B	109.5
C18—C13—H13	105.5	B1—C62—H62C	109.5
C14—C13—H13	105.5	H62A—C62—H62C	109.5
Ti1—C13—H13	63.2	H62B—C62—H62C	109.5
C15—C14—C13	109.65 (9)	C62—B1—C56	106.03 (10)
C15—C14—H14A	109.7	C62—B1—C50	114.14 (9)
C13—C14—H14A	109.7	C56—B1—C50	110.99 (9)
C15—C14—H14B	109.7	C62—B1—C44	109.31 (10)
C13—C14—H14B	109.7	C56—B1—C44	113.91 (9)
H14A—C14—H14B	108.2	C50—B1—C44	102.67 (9)
C16—C15—C14	110.99 (9)	C68A—C63A—C64A	117.2 (4)
C16—C15—H15A	109.4	C68A—C63A—C69A	123.4 (4)
C14—C15—H15A	109.4	C64A—C63A—C69A	119.4 (4)
C16—C15—H15B	109.4	C63A—C64A—C65A	120.2 (5)
C14—C15—H15B	109.4	C63A—C64A—H64A	119.9
H15A—C15—H15B	108.0	C65A—C64A—H64A	119.9
C15—C16—C17	112.15 (9)	C66A—C65A—C64A	120.9 (5)
C15—C16—H16A	109.2	C66A—C65A—H65A	119.5
C17—C16—H16A	109.2	C64A—C65A—H65A	119.5
C15—C16—H16B	109.2	C65A—C66A—C67A	118.9 (4)
C17—C16—H16B	109.2	C65A—C66A—H66A	120.5
H16A—C16—H16B	107.9	C67A—C66A—H66A	120.5
C16—C17—C18	112.67 (10)	C68A—C67A—C66A	119.4 (6)
C16—C17—H17A	109.1	C68A—C67A—H67A	120.3
C18—C17—H17A	109.1	C66A—C67A—H67A	120.3
C16—C17—H17B	109.1	C67A—C68A—C63A	123.3 (6)
C18—C17—H17B	109.1	C67A—C68A—H68A	118.3
H17A—C17—H17B	107.8	C63A—C68A—H68A	118.3
C17—C18—C13	110.28 (9)	C63A—C69A—H69A	109.5
C17—C18—H18A	109.6	C63A—C69A—H69B	109.5
C13—C18—H18A	109.6	H69A—C69A—H69B	109.5
C17—C18—H18B	109.6	C63A—C69A—H69C	109.5
C13—C18—H18B	109.6	H69A—C69A—H69C	109.5
H18A—C18—H18B	108.1	H69B—C69A—H69C	109.5
N2—C19—C20	109.82 (8)	C68B—C63B—C64B	119.3 (4)
N2—C19—C24	113.49 (9)	C68B—C63B—C69B	121.6 (5)
C20—C19—C24	110.77 (9)	C64B—C63B—C69B	119.1 (5)
N2—C19—H19	107.5	C63B—C64B—C65B	118.9 (5)
C20—C19—H19	107.5	C63B—C64B—H64B	120.5
C24—C19—H19	107.5	C65B—C64B—H64B	120.5
C19—C20—C21	111.82 (10)	C66B—C65B—C64B	120.6 (4)
C19—C20—H20A	109.3	C66B—C65B—H65B	119.7
C21—C20—H20A	109.3	C64B—C65B—H65B	119.7
C19—C20—H20B	109.3	C67B—C66B—C65B	120.4 (4)
C21—C20—H20B	109.3	C67B—C66B—H66B	119.8
H20A—C20—H20B	107.9	C65B—C66B—H66B	119.8
C22—C21—C20	110.56 (10)	C66B—C67B—C68B	119.3 (4)
C22—C21—H21A	109.5	C66B—C67B—H67B	120.4
C20—C21—H21A	109.5	C68B—C67B—H67B	120.4
C22—C21—H21B	109.5	C63B—C68B—C67B	121.5 (5)
C20—C21—H21B	109.5	C63B—C68B—H68B	119.3
H21A—C21—H21B	108.1	C67B—C68B—H68B	119.3
C23—C22—C21	110.64 (10)	C63B—C69B—H69D	109.5
C23—C22—H22A	109.5	C63B—C69B—H69E	109.5
C21—C22—H22A	109.5	H69D—C69B—H69E	109.5
C23—C22—H22B	109.5	C63B—C69B—H69F	109.5
C21—C22—H22B	109.5	H69D—C69B—H69F	109.5
H22A—C22—H22B	108.1	H69E—C69B—H69F	109.5
C22—C23—C24	111.61 (10)	C75—C70—C71	119.9 (6)
C22—C23—H23A	109.3	C75—C70—C76	120.0 (6)
C24—C23—H23A	109.3	C71—C70—C76	120.0 (6)
C22—C23—H23B	109.3	C70—C71—C72	120.4 (6)
C24—C23—H23B	109.3	C70—C71—H71	119.8
H23A—C23—H23B	108.0	C72—C71—H71	119.8
C19—C24—C23	111.30 (11)	C71—C72—C73	119.6 (5)
C19—C24—H24A	109.4	C71—C72—H72	120.2
C23—C24—H24A	109.4	C73—C72—H72	120.2
C19—C24—H24B	109.4	C74—C73—C72	118.6 (6)
C23—C24—H24B	109.4	C74—C73—H73	120.7
H24A—C24—H24B	108.0	C72—C73—H73	120.7
N3—C25—C30	113.93 (9)	C73—C74—C75	122.2 (6)
N3—C25—C26	116.25 (9)	C73—C74—H74	118.9
C30—C25—C26	109.20 (10)	C75—C74—H74	118.9
N3—C25—Ti1	44.69 (5)	C74—C75—C70	119.1 (5)
C30—C25—Ti1	118.07 (8)	C74—C75—H75	120.4
C26—C25—Ti1	132.70 (7)	C70—C75—H75	120.4
N3—C25—H25	105.5	C70—C76—H76A	109.5
C30—C25—H25	105.5	C70—C76—H76B	109.5
C26—C25—H25	105.5	H76A—C76—H76B	109.5
Ti1—C25—H25	61.3	C70—C76—H76C	109.5
C27—C26—C25	110.16 (10)	H76A—C76—H76C	109.5
C27—C26—H26A	109.6	H76B—C76—H76C	109.5
C25—C26—H26A	109.6	C71B—C70B—C75B	120.0
C27—C26—H26B	109.6	C71B—C70B—C76B	115.3 (7)
C25—C26—H26B	109.6	C75B—C70B—C76B	124.5 (7)
H26A—C26—H26B	108.1	C72B—C71B—C70B	120.0
C28—C27—C26	111.85 (12)	C72B—C71B—H71B	120.0
C28—C27—H27A	109.2	C70B—C71B—H71B	120.0
C26—C27—H27A	109.2	C71B—C72B—C73B	120.0
C28—C27—H27B	109.2	C71B—C72B—H72B	120.0
C26—C27—H27B	109.2	C73B—C72B—H72B	120.0
H27A—C27—H27B	107.9	C74B—C73B—C72B	120.0
C27—C28—C29	111.67 (12)	C74B—C73B—H73B	120.0
C27—C28—H28A	109.3	C72B—C73B—H73B	120.0
C29—C28—H28A	109.3	C73B—C74B—C75B	120.0
C27—C28—H28B	109.3	C73B—C74B—H74B	120.0
C29—C28—H28B	109.3	C75B—C74B—H74B	120.0
H28A—C28—H28B	107.9	C74B—C75B—C70B	120.0
C28—C29—C30	110.89 (12)	C74B—C75B—H75B	120.0
C28—C29—H29A	109.5	C70B—C75B—H75B	120.0
C30—C29—H29A	109.5	C70B—C76B—H76D	109.5
C28—C29—H29B	109.5	C70B—C76B—H76E	109.5
C30—C29—H29B	109.5	H76D—C76B—H76E	109.5
H29A—C29—H29B	108.1	C70B—C76B—H76F	109.5
C29—C30—C25	110.50 (10)	H76D—C76B—H76F	109.5
C29—C30—H30A	109.6	H76E—C76B—H76F	109.5
			
N2—Ti1—N1—C1	−163.35 (6)	N3—C31—C32B—C33B	−179.6 (6)
N3—Ti1—N1—C1	70.99 (7)	C31—C32B—C33B—C34B	55.8 (10)
N4—Ti1—N1—C1	−43.28 (7)	C32B—C33B—C34B—C35B	−60.0 (11)
C13—Ti1—N1—C1	−130.07 (6)	C33B—C34B—C35B—C36B	56.4 (11)
C25—Ti1—N1—C1	55.24 (10)	N3—C31—C36B—C35B	179.2 (7)
N2—Ti1—N1—C7A	5.40 (14)	C32B—C31—C36B—C35B	49.9 (10)
N3—Ti1—N1—C7A	−120.26 (13)	C34B—C35B—C36B—C31	−50.7 (11)
N4—Ti1—N1—C7A	125.47 (13)	C43—C38—C39—C40	1.09 (17)
C13—Ti1—N1—C7A	38.68 (13)	C37—C38—C39—C40	−177.54 (11)
C25—Ti1—N1—C7A	−136.01 (12)	C38—C39—C40—C41	0.13 (17)
C1—Ti1—N1—C7A	168.75 (17)	C39—C40—C41—F1	178.23 (10)
N2—Ti1—N1—C7B	21.1 (2)	C39—C40—C41—C42	−1.09 (19)
N3—Ti1—N1—C7B	−104.60 (19)	F1—C41—C42—C43	−178.53 (11)
N4—Ti1—N1—C7B	141.13 (19)	C40—C41—C42—C43	0.8 (2)
C13—Ti1—N1—C7B	54.34 (19)	C41—C42—C43—C38	0.5 (2)
C25—Ti1—N1—C7B	−120.4 (2)	C39—C38—C43—C42	−1.39 (19)
C1—Ti1—N1—C7B	−175.6 (2)	C37—C38—C43—C42	177.24 (12)
N1—Ti1—N2—C19	−98.56 (12)	C49—C44—C45—F2	177.74 (10)
N3—Ti1—N2—C19	28.29 (13)	B1—C44—C45—F2	2.28 (17)
N4—Ti1—N2—C19	144.67 (11)	C49—C44—C45—C46	−0.48 (16)
C13—Ti1—N2—C19	−175.81 (15)	B1—C44—C45—C46	−175.93 (11)
C25—Ti1—N2—C19	60.26 (12)	F2—C45—C46—F3	1.49 (16)
C1—Ti1—N2—C19	−112.40 (11)	C44—C45—C46—F3	179.79 (10)
N1—Ti1—N2—C13	77.25 (7)	F2—C45—C46—C47	−178.49 (10)
N3—Ti1—N2—C13	−155.90 (6)	C44—C45—C46—C47	−0.19 (18)
N4—Ti1—N2—C13	−39.52 (7)	F3—C46—C47—F4	0.44 (17)
C25—Ti1—N2—C13	−123.92 (6)	C45—C46—C47—F4	−179.58 (10)
C1—Ti1—N2—C13	63.42 (8)	F3—C46—C47—C48	−179.43 (11)
N2—Ti1—N3—C31	−111.07 (12)	C45—C46—C47—C48	0.55 (17)
N1—Ti1—N3—C31	13.69 (13)	F4—C47—C48—F5	0.04 (18)
N4—Ti1—N3—C31	128.65 (12)	C46—C47—C48—F5	179.90 (11)
C13—Ti1—N3—C31	−132.13 (11)	F4—C47—C48—C49	179.92 (11)
C25—Ti1—N3—C31	177.33 (16)	C46—C47—C48—C49	−0.21 (18)
C1—Ti1—N3—C31	44.58 (13)	F5—C48—C49—F6	−2.23 (17)
N2—Ti1—N3—C25	71.60 (7)	C47—C48—C49—F6	177.89 (11)
N1—Ti1—N3—C25	−163.64 (6)	F5—C48—C49—C44	179.35 (11)
N4—Ti1—N3—C25	−48.68 (7)	C47—C48—C49—C44	−0.53 (19)
C13—Ti1—N3—C25	50.54 (9)	C45—C44—C49—F6	−177.51 (10)
C1—Ti1—N3—C25	−132.75 (6)	B1—C44—C49—F6	−1.66 (16)
C7A—N1—C1—C2	57.47 (12)	C45—C44—C49—C48	0.84 (17)
C7B—N1—C1—C2	44.1 (3)	B1—C44—C49—C48	176.69 (11)
Ti1—N1—C1—C2	−129.83 (8)	C55—C50—C51—F7	176.95 (9)
C7A—N1—C1—C6	−70.28 (11)	B1—C50—C51—F7	−6.44 (15)
C7B—N1—C1—C6	−83.6 (3)	C55—C50—C51—C52	−0.84 (15)
Ti1—N1—C1—C6	102.43 (8)	B1—C50—C51—C52	175.77 (10)
C7A—N1—C1—Ti1	−172.71 (11)	F7—C51—C52—F8	0.74 (14)
C7B—N1—C1—Ti1	173.9 (3)	C50—C51—C52—F8	178.61 (10)
N1—C1—C2—C3	172.23 (9)	F7—C51—C52—C53	−178.01 (9)
C6—C1—C2—C3	−57.87 (12)	C50—C51—C52—C53	−0.15 (16)
Ti1—C1—C2—C3	123.94 (10)	F8—C52—C53—F9	2.71 (15)
C1—C2—C3—C4	56.57 (13)	C51—C52—C53—F9	−178.52 (9)
C2—C3—C4—C5	−54.83 (15)	F8—C52—C53—C54	−177.42 (9)
C3—C4—C5—C6	54.97 (14)	C51—C52—C53—C54	1.35 (16)
C4—C5—C6—C1	−57.95 (12)	F9—C53—C54—F10	−1.28 (16)
N1—C1—C6—C5	−169.71 (9)	C52—C53—C54—F10	178.85 (9)
C2—C1—C6—C5	59.22 (12)	F9—C53—C54—C55	178.36 (10)
Ti1—C1—C6—C5	−122.18 (9)	C52—C53—C54—C55	−1.51 (16)
C1—N1—C7A—C8A	93.63 (12)	F10—C54—C55—F11	0.06 (15)
Ti1—N1—C7A—C8A	−74.64 (17)	C53—C54—C55—F11	−179.57 (10)
C1—N1—C7A—C12A	−143.00 (10)	F10—C54—C55—C50	−179.88 (10)
Ti1—N1—C7A—C12A	48.73 (17)	C53—C54—C55—C50	0.48 (17)
N1—C7A—C8A—C9A	−176.89 (11)	C51—C50—C55—F11	−179.27 (9)
C12A—C7A—C8A—C9A	59.79 (15)	B1—C50—C55—F11	4.39 (17)
C7A—C8A—C9A—C10A	−58.39 (16)	C51—C50—C55—C54	0.67 (16)
C8A—C9A—C10A—C11A	55.09 (17)	B1—C50—C55—C54	−175.67 (10)
C9A—C10A—C11A—C12A	−53.50 (18)	C61—C56—C57—F12	178.88 (10)
C10A—C11A—C12A—C7A	55.42 (18)	B1—C56—C57—F12	−8.89 (18)
N1—C7A—C12A—C11A	175.32 (11)	C61—C56—C57—C58	−1.40 (17)
C8A—C7A—C12A—C11A	−58.38 (16)	B1—C56—C57—C58	170.84 (11)
C1—N1—C7B—C12B	−70.1 (5)	F12—C57—C58—F13	−1.23 (17)
Ti1—N1—C7B—C12B	103.4 (4)	C56—C57—C58—F13	179.03 (11)
C1—N1—C7B—C8B	53.7 (4)	F12—C57—C58—C59	179.03 (11)
Ti1—N1—C7B—C8B	−132.8 (3)	C56—C57—C58—C59	−0.71 (19)
N1—C7B—C8B—C9B	177.0 (4)	F13—C58—C59—F14	0.99 (19)
C12B—C7B—C8B—C9B	−57.6 (5)	C57—C58—C59—F14	−179.26 (11)
C7B—C8B—C9B—C10B	54.3 (6)	F13—C58—C59—C60	−177.97 (12)
C8B—C9B—C10B—C11B	−55.4 (6)	C57—C58—C59—C60	1.77 (19)
C9B—C10B—C11B—C12B	56.8 (6)	F14—C59—C60—F15	0.0 (2)
N1—C7B—C12B—C11B	179.9 (4)	C58—C59—C60—F15	178.92 (12)
C8B—C7B—C12B—C11B	58.9 (5)	F14—C59—C60—C61	−179.60 (12)
C10B—C11B—C12B—C7B	−57.0 (6)	C58—C59—C60—C61	−0.6 (2)
C19—N2—C13—C18	41.17 (13)	F15—C60—C61—F16	−2.17 (18)
Ti1—N2—C13—C18	−135.80 (8)	C59—C60—C61—F16	177.37 (12)
C19—N2—C13—C14	−87.03 (11)	F15—C60—C61—C56	178.73 (12)
Ti1—N2—C13—C14	96.00 (8)	C59—C60—C61—C56	−1.7 (2)
C19—N2—C13—Ti1	176.97 (11)	C57—C56—C61—F16	−176.43 (11)
N2—C13—C14—C15	−167.02 (8)	B1—C56—C61—F16	10.61 (17)
C18—C13—C14—C15	61.83 (11)	C57—C56—C61—C60	2.62 (18)
Ti1—C13—C14—C15	−117.96 (8)	B1—C56—C61—C60	−170.33 (12)
C13—C14—C15—C16	−58.63 (12)	C57—C56—B1—C62	−116.23 (13)
C14—C15—C16—C17	52.97 (14)	C61—C56—B1—C62	55.55 (14)
C15—C16—C17—C18	−50.98 (14)	C57—C56—B1—C50	8.24 (16)
C16—C17—C18—C13	54.19 (13)	C61—C56—B1—C50	−179.98 (10)
N2—C13—C18—C17	169.59 (9)	C57—C56—B1—C44	123.52 (12)
C14—C13—C18—C17	−59.24 (11)	C61—C56—B1—C44	−64.70 (13)
Ti1—C13—C18—C17	120.47 (10)	C55—C50—B1—C62	2.65 (16)
C13—N2—C19—C20	−159.47 (9)	C51—C50—B1—C62	−173.45 (10)
Ti1—N2—C19—C20	15.87 (16)	C55—C50—B1—C56	−117.09 (11)
C13—N2—C19—C24	75.93 (11)	C51—C50—B1—C56	66.81 (13)
Ti1—N2—C19—C24	−108.72 (12)	C55—C50—B1—C44	120.82 (11)
N2—C19—C20—C21	178.77 (9)	C51—C50—B1—C44	−55.28 (12)
C24—C19—C20—C21	−55.08 (12)	C45—C44—B1—C62	−127.63 (12)
C19—C20—C21—C22	56.96 (13)	C49—C44—B1—C62	57.19 (13)
C20—C21—C22—C23	−57.11 (14)	C45—C44—B1—C56	−9.25 (16)
C21—C22—C23—C24	56.54 (16)	C49—C44—B1—C56	175.57 (10)
N2—C19—C24—C23	177.70 (9)	C45—C44—B1—C50	110.84 (12)
C20—C19—C24—C23	53.62 (13)	C49—C44—B1—C50	−64.34 (12)
C22—C23—C24—C19	−54.90 (15)	C68A—C63A—C64A—C65A	−0.1 (9)
C31—N3—C25—C30	−71.73 (12)	C69A—C63A—C64A—C65A	−179.4 (6)
Ti1—N3—C25—C30	106.47 (8)	C63A—C64A—C65A—C66A	−0.4 (10)
C31—N3—C25—C26	56.59 (12)	C64A—C65A—C66A—C67A	−0.4 (9)
Ti1—N3—C25—C26	−125.22 (8)	C65A—C66A—C67A—C68A	1.7 (10)
C31—N3—C25—Ti1	−178.20 (11)	C66A—C67A—C68A—C63A	−2.3 (11)
N3—C25—C26—C27	170.62 (10)	C64A—C63A—C68A—C67A	1.5 (9)
C30—C25—C26—C27	−58.79 (13)	C69A—C63A—C68A—C67A	−179.3 (6)
Ti1—C25—C26—C27	119.21 (11)	C68B—C63B—C64B—C65B	−0.7 (7)
C25—C26—C27—C28	56.33 (15)	C69B—C63B—C64B—C65B	−179.2 (5)
C26—C27—C28—C29	−53.89 (17)	C63B—C64B—C65B—C66B	0.4 (7)
C27—C28—C29—C30	54.14 (17)	C64B—C65B—C66B—C67B	0.0 (6)
C28—C29—C30—C25	−57.47 (15)	C65B—C66B—C67B—C68B	0.1 (7)
N3—C25—C30—C29	−168.38 (10)	C64B—C63B—C68B—C67B	0.8 (6)
C26—C25—C30—C29	59.79 (13)	C69B—C63B—C68B—C67B	179.2 (5)
Ti1—C25—C30—C29	−118.54 (10)	C66B—C67B—C68B—C63B	−0.4 (7)
C25—N3—C31—C36B	122.9 (5)	C75—C70—C71—C72	2.3 (8)
Ti1—N3—C31—C36B	−54.2 (5)	C76—C70—C71—C72	179.2 (6)
C25—N3—C31—C32B	−107.3 (4)	C70—C71—C72—C73	−0.9 (8)
Ti1—N3—C31—C32B	75.7 (4)	C71—C72—C73—C74	0.9 (8)
C25—N3—C31—C36A	88.91 (13)	C72—C73—C74—C75	−2.5 (9)
Ti1—N3—C31—C36A	−88.19 (15)	C73—C74—C75—C70	3.9 (10)
C25—N3—C31—C32A	−148.66 (12)	C71—C70—C75—C74	−3.8 (9)
Ti1—N3—C31—C32A	34.25 (18)	C76—C70—C75—C74	179.3 (6)
N3—C31—C32A—C33A	175.77 (15)	C75B—C70B—C71B—C72B	0.0
C36A—C31—C32A—C33A	−58.4 (2)	C76B—C70B—C71B—C72B	−175.8 (8)
C31—C32A—C33A—C34A	56.2 (3)	C70B—C71B—C72B—C73B	0.0
C32A—C33A—C34A—C35A	−53.3 (3)	C71B—C72B—C73B—C74B	0.0
C33A—C34A—C35A—C36A	54.5 (2)	C72B—C73B—C74B—C75B	0.0
C34A—C35A—C36A—C31	−58.33 (18)	C73B—C74B—C75B—C70B	0.0
N3—C31—C36A—C35A	−176.97 (12)	C71B—C70B—C75B—C74B	0.0
C32A—C31—C36A—C35A	59.13 (16)	C76B—C70B—C75B—C74B	175.4 (9)
C36B—C31—C32B—C33B	−51.7 (9)		

### Tris(dicyclohexylamido)(4-fluorobenzonitrile)titanium methyltris(pentafluorophenyl)borate toluene sesquisolvate (2) Hydrogen-bond geometry (Å, º)

**Table:**

*D*—H···*A*	*D*—H	H···*A*	*D*···*A*	*D*—H···*A*
C62—H62*A*···F6	0.98	2.35	2.9753 (17)	121
C62—H62*C*···F16	0.98	2.38	2.9931 (16)	120
C9*A*—H9*AB*···F2^i^	0.99	2.53	3.1938 (17)	124
C71*B*—H71*B*···F14^i^	0.95	2.58	3.176 (5)	121
C17—H17*B*···F4^ii^	0.99	2.50	3.2834 (15)	136
C34*A*—H34*A*···F16	0.99	2.45	3.296 (2)	143
C43—H43···F8^iii^	0.95	2.55	3.2229 (13)	128
C75—H75···F14^iv^	0.95	2.47	3.212 (6)	135
C39—H39···F11^v^	0.95	2.47	3.3904 (13)	163
C40—H40···F6^v^	0.95	2.43	3.3413 (13)	160
C66*B*—H66*B*···F11^iii^	0.95	2.49	3.372 (4)	154
C5—H5*B*···F10^v^	0.99	2.52	3.4028 (14)	149
C16—H16*B*···F9^iii^	0.99	2.56	3.4280 (14)	147

Symmetry codes: (i) *x*+1, *y*, *z*; (ii) −*x*+1, −*y*+1, −*z*+1; (iii) *x*+1, *y*−1, *z*; (iv) −*x*, −*y*+2, −*z*; (v) *x*, *y*−1, *z*.
